# STING agonists trigger monocyte death via apoptosis, pyroptosis, caspase-8 activation and mitochondrial dysfunction

**DOI:** 10.1038/s41420-025-02786-1

**Published:** 2025-10-31

**Authors:** Marketa Pimkova Polidarova, Lydie Plecita-Hlavata, Ivan Hirsch, Klara Grantz Saskova, Andrea Brazdova

**Affiliations:** 1 Department of Genetics and Microbiology, Faculty of Science, Charles University, BIOCEV, Vestec, Czech Republic; 2 Institute of Organic Chemistry and Biochemistry of the Czech Academy of Sciences, Prague, Czech Republic; 3 Laboratory of Pancreatic Islet Research, Institute of Physiology of the Czech Academy of Sciences, Prague, Czech Republic

**Keywords:** Cell death and immune response, Cell death

## Abstract

The cyclic GMP–AMP synthase–stimulator of interferon genes (cGAS–STING) pathway senses double-stranded DNA in the cytoplasm, triggering the secretion of type I and III interferons and proinflammatory cytokines. However, cGAS–STING pathway activation by STING agonists also induces regulated cell death (RCD) in human monocytes, which is inherently linked to cytokine production. We identified that STING agonist-induced monocyte RCD integrates apoptotic (active caspase-9, -8 and -3/7) and pyroptotic mechanisms (active caspase-1, cleaved gasdermin-D and secreted mature interleukin-1β and -18), whereas necroptosis is inhibited through caspase-8-mediated cleavage of receptor-interacting protein kinase 1 (RIPK1). Additionally, this RCD is accompanied by mitochondrial dysfunction, which precedes caspase activation, suggesting that mitochondrial disruption may act as both the driving mechanism and a direct outcome of cGAS–STING pathway activation via the STING–IRF3–BAX pathway. Overall, this study identifies a novel RCD process that may be described as ‘pyroptotic apoptosis’, providing new insights into the outcomes of cGAS–STING signalling in human monocytes.

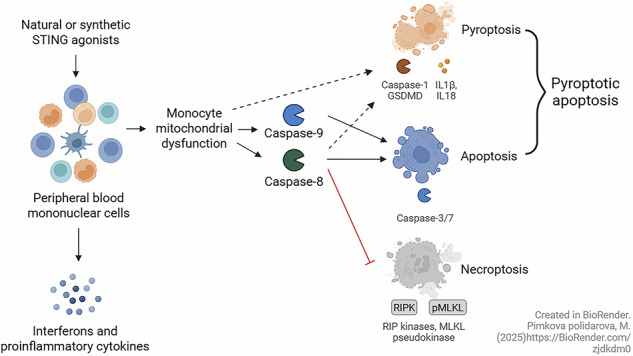

## Facts

- STING agonists induce the secretion of a unique cytokine signature in peripheral blood mononuclear cells, distinct from that triggered by toll-like receptor agonists.
- Unlike toll-like receptor agonists, treatment with STING agonists leads to the near-complete depletion of non-classical, intermediate and classical monocytes due to cell death, accompanied by the loss of CD14 and CD16 markers.
- STING agonist-induced monocyte cell death integrates both apoptotic (active caspase-9, -8, -3 and -7) and pyroptotic (active caspase-1, cleaved gasdermin-D, and secreted interleukin-1β and -18) mechanisms, whereas necroptosis is inhibited by caspase-8-mediated cleavage of receptor-interacting protein kinase 1. This process can be described as ‘apoptotic pyroptosis’.
- Mitochondrial dysfunction precedes caspase activation, suggesting potential mitochondrial regulation of STING agonist-induced monocyte cell death.

## Questions

- What is the mechanism of STING agonists-induced monocyte ‘apoptotic pyroptosis’? Is monocyte cell death regulated by mitochondria?
- How is pyroptosis activated? Is it regulated by mitochondria or independently?
- Do STING agonists induce similar processes in other immune subsets, especially T cells, NK cells or B cells, assuming time-dependent effects? What would be the direct outcomes of cGAS–STING signaling and the results of immune crosstalk?
- Could STING agonist-induced monocyte RCD be exploited for therapeutic purposes, for example, in STING-associated disorders or antitumour therapy?

## Introduction

Regulated cell death (RCD) plays a vital role in organism development, homeostasis and immune system function. Key RCD pathways include apoptosis, pyroptosis and necroptosis [1]. Apoptosis is typically an immunologically silent death where cells shrink and form apoptotic bodies, which are cleared by phagocytosis [1]. This process is mediated by caspases activated either extrinsically via plasma membrane receptors or intrinsically via mitochondria, in both cases leading to the activation of effector caspase-3 and -7 [1, 2].

Unlike apoptosis, pyroptosis and necroptosis involve plasma membrane pore formation and the release of damage-associated molecular patterns (DAMPs), resulting in immune activation. Pyroptosis is driven by inflammasomes, where active caspases (-1 or -4/5 in humans) proteolytically cleave gasdermins (e.g., gasdermin-D—GSDMD), triggering their pore-forming activity [1, 3]. Additionally, the caspase-1 inflammasome processes the proinflammatory cytokines interleukin-1β (IL1β) and IL18 into their mature forms; they then get secreted through GSDMD pores, along with DAMPs [1, 3, 4]. Unlike apoptosis and pyroptosis, necroptosis is executed by receptor-interacting protein kinases (RIPK) 1 and 3, which activate mixed lineage kinase domain-like pseudokinase (MLKL). MLKL then forms plasma membrane pores, disrupting the ion balance between the intra- and extracellular environments, resulting in cell rupture and DAMP release [1].

RCD pathways form a complex network [1]. Extrinsic apoptosis initiator caspase-8 can trigger intrinsic apoptosis in certain cell types [1, 2, 5], inhibit necroptosis [2, 6] and activate pyroptosis [7, 8]. Moreover, PANoptosis has recently been described in influenza A virus infection [9], combining aspects of pyroptosis, apoptosis and necroptosis. Multiple PANoptosomes have been reported in the context of viral and bacterial infections, highlighting the importance of PANoptosis in immune defense against pathogens [10, 11].

Importantly, pathogens and intrinsic cell damage are detected by pattern-recognition receptors (PRR), among which nucleic acid sensors are critical for the recognition of intracellular pathogens [12]. PRR activation typically triggers cytokine secretion, but it can also induce RCD [13]. Among PRR pathways, the sensor of cytoplasmic double-stranded DNA (dsDNA), cyclic GMP–AMP synthase–stimulator of interferon genes (cGAS–STING) pathway [14], is of particular interest as a driver of immunogenic RCD and antitumour immunity [15]. Upon dsDNA binding, cGAS synthesizes the cyclic dinucleotide (CDN) 2′,3′-cGAMP, which binds to STING, further activating a signaling cascade that leads to proinflammatory cytokine production [16]. STING can also be directly activated by bacterial CDNs, synthetic compounds [17–19], other dsDNA sensors [20] or endoplasmic reticulum stress [14, 21, 22].

We previously investigated the activation of the cGAS–STING pathway in peripheral blood mononuclear cells (PBMCs) [17–19]. Apart from the secretion of a broad portfolio of proinflammatory cytokines, STING agonists induced a complete depletion of the CD14+ monocyte population due to RCD of apoptotic characteristics [19]. Both monocyte death and interferon α (IFNα) secretion were blocked by TBK1 inhibition, demonstrating their dependence on cGAS–STING pathway activation. Moreover, the cGAS–STING pathway has been implicated in multiple RCD pathways triggered in response to various stimuli in both mice and humans [23–26].

As monocytes play a crucial role in innate immune surveillance and the initiation of immune responses [27], we further explored the mechanisms of STING agonist-induced monocyte RCD. We investigated activation of apoptotic, pyroptotic and necroptotic processes in human monocytes and the role of mitochondria in monocyte RCD. Combining the obtained results, we proposed a possible model for STING agonist-induced RCD in human monocytes. As cGAS–STING pathway activation is of therapeutic interest, our findings provide new insights into the effects of STING agonists relevant to STING-targeting therapeutic strategies.

## Results

### STING agonists induce the secretion of a unique cytokine signature in human PBMCs

**Fig. 1:**
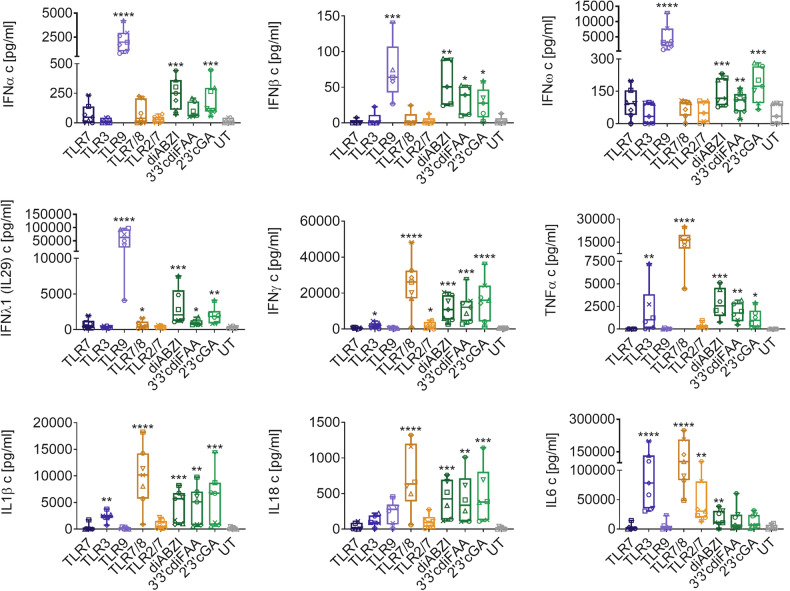
STING agonists induce the secretion of a unique cytokine portfolio. Human PBMCs (*n* = 5–7 donors) were treated for 16 h with TLR agonists: TLR7 agonist GS-9620, TLR3 agonist poly I:C (HMW), TLR9 agonist CpG-A (ODN2216), TLR7/8 agonist Resiquimod, TLR2/7 agonist Adilipoline, and STING agonists: diaminobenzimidazole-based agonist (diABZI), 3′,3′c-di(2′F,2′d-AMP) (3′3′cdiFAA) and 2′,3′-cGAMP (2′3′cGA). Untreated cells (UT) were used as control samples. The concentration of secreted cytokines in the medium was multiplexed using ProcartaPlex Immunoassays (type I interferons, IFNs: IFNα, IFNβ, IFNω; type II IFN: IFNγ; type III IFN: IFNλ1 (IL29); proinflammatory cytokines: tumor necrosis factor alpha (TNFα), interleukin-1β (IL1β), IL18, IL6). Data were analyzed using the Friedman test with uncorrected Dunn’s test for multiple comparisons. Individual donors are represented by different symbols. *P < 0.05, **P < 0.01, ***P < 0.001, ****P < 0.0001.

We first analyzed the cytokine profile secreted by human PBMCs in response to various PRR pathway agonists [17–19, 28]. To demonstrate the unique properties of cGAS-STING pathway activation, we compared selected Toll-like receptor (TLR) agonists targeting nucleic acid-sensing TLRs: the TLR7 agonist GS-9620, the TLR3 agonist poly I:C (HMW) and the TLR9 agonist CpG-A (ODN2216); two dual-acting TLR agonists: the TLR7/8 agonist Resiquimod and the TLR2/7 agonist Adilipoline; and three selected STING agonists with distinct properties: the lipophilic, synthetic, diaminobenzimidazole-based agonist (diABZI); the polar, synthetic CDN-based STING agonist 3′,3′c-di(2′F,2′d-AMP) (3′3′cdiFAA); and the polar, natural eukaryotic CDN-based STING agonist 2′,3′-cGAMP (2′3′cGA). Based on the secreted cytokine signature, TLR agonists were categorized into two groups: type I and III IFN inducers (TLR9 and TLR7 agonists); and proinflammatory cytokine inducers (TLR7/8 >TLR3 >TLR2/7 agonists, Fig. 1). The TLR9 agonist was the strongest activator of type I and III IFNs due to pDC activation [29] whereas the TLR7 agonist induced IFNα, ω and IFNλ1 (IL29) non-significantly (Fig. 1). The level of cytokine secretion induced by the TLR7 agonist was weaker than in our previous report [28]. Additionally, the TLR7/8 agonist also weakly stimulated IFNλ1 (IL29) and non-significantly stimulated IFNα and IFNω. Overall, the cytokine signatures of the tested TLR agonists were consistent with and expanded upon our previously published results [28]. Importantly, only STING agonists triggered a unique cytokine combination of type I, II and III IFNs, along with proinflammatory cytokines (Fig. 1).

### STING agonists induce robust depletion of all monocyte subsets

**Fig. 2:**
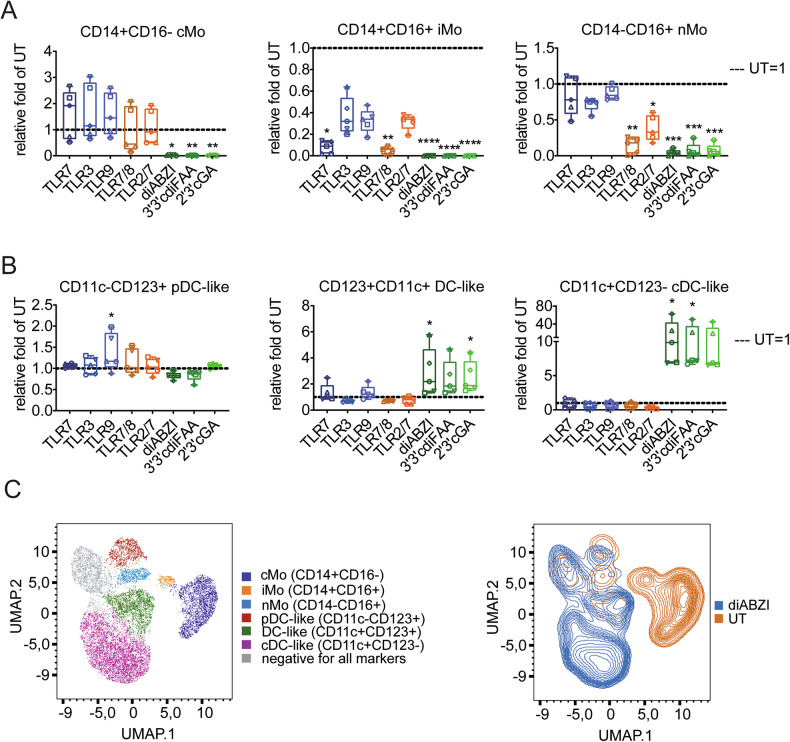
STING agonists induce almost complete depletion of monocyte populations in PBMCs. Human PBMCs were treated for 16 h with TLR agonists: TLR7 agonist GS-9620, TLR3 agonist poly I:C (HMW), TLR9 agonist CpG-A (ODN2216), TLR7/8 agonist Resiquimod, TLR2/7 agonist Adilipoline, and STING agonists: diaminobenzimidazole-based agonist (diABZI), 3′,3′c-di(2′F,2′d-AMP) (3′3′cdiFAA), and 2′,3′-cGAMP (2′3′cGA). Untreated cells (UT) were used as control samples. The frequency of immune populations was analyzed using multiplex immunophenotyping flow cytometry-based analyses. **A**, **B** The effect on immune subsets was analyzed as the relative fold difference in the frequency of the respective population between treated samples and UT (UT = 1, dashed lines). Individual donors are represented by different symbols. Data were analyzed with the Friedman test with the uncorrected Dunn’s test for multiple comparisons. *P < 0.05, **P < 0.01, ***P < 0.001, ****P < 0.0001. **A** Effect on monocyte (Mo) populations (*n =* 5 PBMC donors): CD14+CD16- classical Mo (cMo), CD14+CD16+ intermediate Mo (iMo), CD14-CD16+ non-classical Mo (nMo). **B** Effect on dendritic cell (DC)-like populations (*n* = 5 PBMC donors): CD11c-CD123+ plasmacytoid DC (pDC)-like, CD11c+CD123+ DC-like, CD11c+CD123- conventional DC (cDC)-like cells. **C** Representative example (*n* = 1 PBMC donor) of unbiased multiparametric cluster analyses of the myeloid population of PBMCs and the effect of diABZI (a representative STING agonist) on immune subsets.

Next, we investigated the effects of PRR activators on PBMC immune subsets to further expand on our previous findings [19]. TLR7 and TLR7/8 agonists significantly depleted intermediate monocytes (iMo), while TLR3, TLR9 and TLR2/7 agonists non-significantly reduced iMo levels to below 50% (Fig. 2A). The effect of TLR agonists on classical monocytes (cMo) varied among individual donors, whereas non-classical monocytes (nMo) were depleted upon TLR7/8 and TLR2/7 agonist treatment. Uniquely, STING agonists caused a near-complete depletion of classical, intermediate and non-classical monocytes, again demonstrating the cGAS-STING pathway activation-specific effects (Fig. 2A).

We next assessed whether the depleting monocytes differentiated into dendritic cells (DCs). The STING agonist treatment enriched conventional (cDC)-like and CD11c+CD123+ DC-like [30] populations (Fig. 2B), despite considerable data dispersion caused by donor-to-donor variability in cDC-like frequency in untreated cells (UT; Fig. 2B). However, the enriched population originated from depleting monocytes undergoing cell death (Fig. S1A), with concurrent loss of CD14 [19] and CD16, rather than from the differentiation of monocytes into DCs. Among TLR agonists, only TLR9 activation significantly enriched the pDC-like population (Fig. 2B).

To better understand the effects of STING agonists on myeloid PBMC populations (‘lin- myelo’, Fig. S2), we performed unbiased multiparametric analyses. These revealed substantial immunophenotypic changes, including monocyte loss accompanied by phenotypic enrichment of DC-like populations, and the emergence of cells lacking the analyzed markers (Fig. 2C).

Finally, we examined the effects of PRR activators on lymphoid subsets (Fig. S1B). STING agonists had no significant impact on T, B, NK or NKT cell frequencies. However, the TLR7/8 and TLR2/7 agonists enriched T cells, while the TLR9 agonist significantly enriched NKT cells.

### Monocytes, within the PBMC context, respond to the STING agonist treatment by activating apoptotic and pyroptotic caspases

**Fig. 3:**
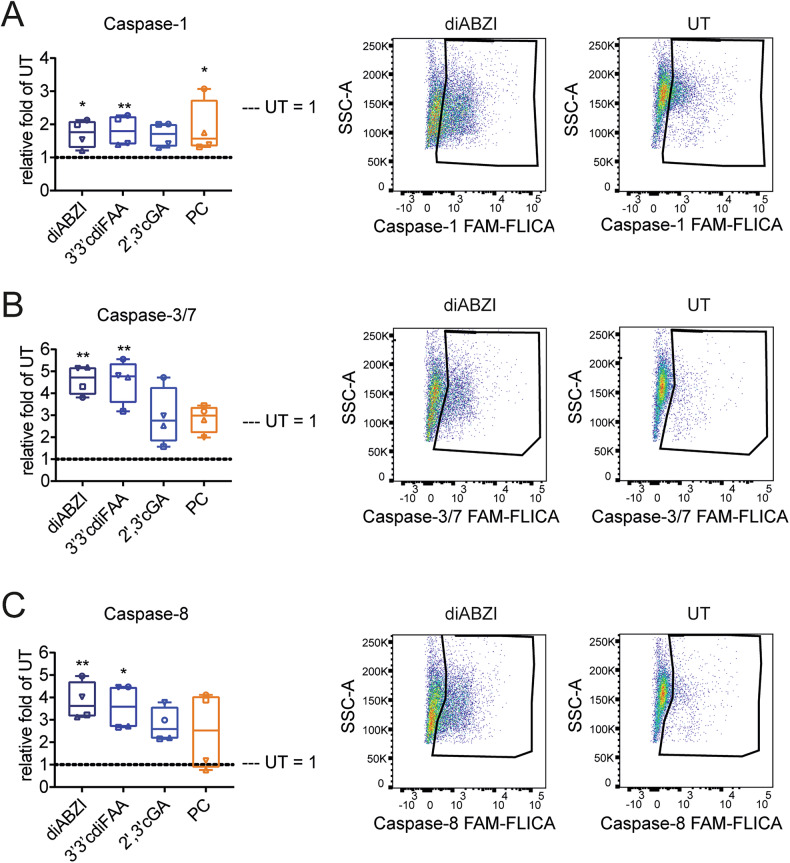
STING agonists induce the activation of apoptotic and pyroptotic caspases in the myeloid subset of PBMCs. Human PBMCs (*n* = 4 donors) were treated for 4 h with STING agonists: diaminobenzimidazole-based agonist (diABZI), 3′,3′c-di(2′F,2′d-AMP) (3′3′cdiFAA) and 2′,3′-cGAMP (2′3′cGA). Untreated cells (UT) were used as a negative control. The frequency of immune populations was analyzed using multiplex immunophenotyping flow cytometry-based analyses. Active caspases were detected using FAM-FLICA assays specific to each respective caspase. The effect on immune subsets and caspase activation was analyzed as the relative fold difference in the frequency of the respective population between treated samples and UT (UT = 1, dashed lines). Individual donors are represented by different symbols. Graphs are accompanied by representative examples of activated caspases in the myeloid population. Data were analyzed with the Friedman test with the uncorrected Dunn’s test for multiple comparisons. *P < 0.05, **P < 0.01. **A** Activation of caspase-1. Positive control (PC) = nigericin. **B** Activation of caspases-3/7. PC staurosporine. **C** Activation of caspase-8. PC staurosporine.

Because STING agonists exhibited distinct properties from TLRs, we further examined the mechanisms behind STING agonist-induced monocyte depletion. As we previously described [19], monocyte death occurred rapidly within 4 h after treatment. We first investigated the roles of apoptosis and pyroptosis, marked by active caspases-3 and -7 (caspase-3/7) and caspase-1, respectively. Additionally, we included caspase-8, which can participate in apoptosis, pyroptosis, necroptosis and PANoptosis [2, 6]. Given the loss of CD14 and CD16 markers from monocytes within 4 h of STING agonist treatment (Fig. S3A), we gated the PBMC myeloid subset for further analysis (‘lin- myelo’, Fig. S4). Using FAM-FLICA-based detection of active caspases, we demonstrated that STING agonists activated all the analyzed caspases (Fig. 3). Additionally, most caspase-positive cells were live/dead marker-negative, indicating an early stage of RCD, although live/dead marker-positive cells were also enriched, regardless of their caspase positivity (Fig. S3B).

### STING agonists activate apoptotic and pyroptotic caspases in enriched monocytes, while necroptosis is inhibited

**Fig. 4:**
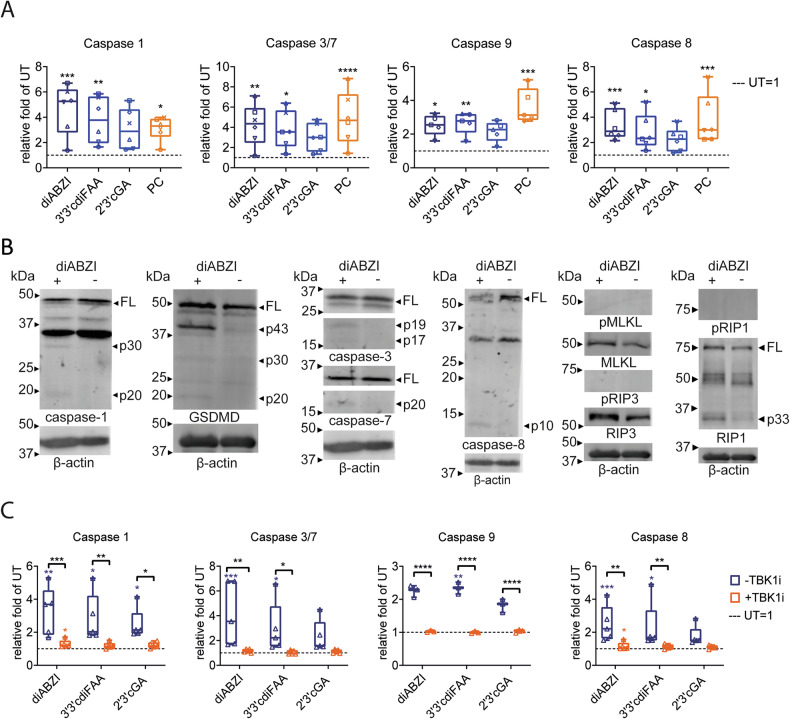
STING agonists induce the activation of apoptotic and pyroptotic caspases in enriched monocytes. **A** Monocytes were enriched from PBMCs (*n* = 5–6 donors) and treated for 4 h with STING agonists: diaminobenzimidazole-based agonist (diABZI), 3′,3′c-di(2′F,2′d-AMP) (3′3′cdiFAA) and 2′,3′-cGAMP (2′3′cGA). Positive control (PC) = nigericin or staurosporine was used for caspase-1, or caspases-3/7, -8, and -9, respectively. Untreated cells (UT) were used as a negative control. Activation of caspases-1, -3/7, -8, and -9 was analyzed using reporter-based assays. The data are presented as the relative fold difference in relative luminescence units between treated samples and UT (UT = 1, dashed lines). Individual donors are represented by different symbols. Data were analyzed with the Friedman test with the uncorrected Dunn’s test for multiple comparisons. *P < 0.05, **P < 0.01, ***P < 0.001, ****P < 0.0001. **B** Western blot-based immunodetection was used to verify the presence of activated caspases in monocytes and broaden the analysis to gasdermin D (GSDMD-D) and necroptotic pathway proteins (RIP1, RIP3, MLKL and their phosphorylated forms). β-actin was used as a loading control. Monocytes were enriched from PBMCs and treated for 4 h with diABZI (only one STING agonist was selected because of the method’s sample requirements). Untreated cells (UT) were used as a negative control. Representative composition of immunodetection of regulated cell death factors (full-length membranes of 2–4 PBMC donors can be found in the Supporting Information file WB_supplementary.pdf’). FL full length. **C** To demonstrate that the caspase activation was dependent on cGAS–STING pathway activation, enriched monocytes (*n* = 3–5 PBMC donors) were pretreated for 30 min with TBK1 inhibitor (TBK1i) MRT68601 hydrochloride before 4 h of STING agonist stimulation (+TBK1i): diaminobenzimidazole-based agonist (diABZI), 3′,3′c-di(2′F,2′d-AMP) (3′3′cdiFAA) and 2′,3′-cGAMP (2′3′cGA). Cells without TBK1i pretreatment (−TBK1i) were used for comparison. Untreated cells (UT) were used as a negative control in both +TBK1i and −TBK1i conditions. A reporter-based assay was used to measure caspase activation. The data are presented as the relative fold difference in relative luminescence units between treated samples to UT belonging to the respective −/+TBK1i groups (blue = −TBK1i, orange = +TBK1i) (UT = 1, dashed line). The data were analyzed with two-way ANOVA to compare −TBK1i and +TBK1i and with the Friedman test with the uncorrected Dunn’s test for multiple comparisons to compare differences against the respective UT. *P < 0.05, **P < 0.01, ***P < 0.001, ****P < 0.0001.

To confirm that the observed caspase activation was a direct effect of STING agonists on monocytes, rather than an indirect outcome of crosstalk among individual PBMC subsets, we analyzed the effect of STING agonists on enriched monocytes. Consistent with our PBMC results (Fig. 3), STING agonists activated all the analyzed caspases in enriched monocytes, including caspase-9, which is an initiator of the mitochondrial apoptotic pathway (Fig. 4A). Importantly, the caspase-1 reporter assay may also detect the activation of pyroptotic caspase-5 and apoptotic caspase-3 and -6 [31]. Caspase-1 inhibition reduced but did not eliminate other caspase activity, suggesting the activation of cross-reactive caspases (Fig. S5A). However, we did not further investigate whether this residual activity could solely be attributed to caspase-3.

Western blot analysis further confirmed the presence of active caspases in monocytes treated with the STING agonist diABZI (Fig. 4B). Interestingly, we detected not only a fully cleaved 20-kDa fragment of caspase-1 (albeit weakly in cells from some donors), but also a 33-kDa cleavage intermediate, which was identified as an inflammasome effector [32]. Additionally, we analyzed GSDMD, a pore-forming protein activated in pyroptosis, and detected cleaved GSDMD forms of sizes corresponding to processing by caspase-1 and -3/7 (Fig. 4B) [7, 33], further confirming the activation of both pyroptotic and apoptotic pathways.

Furthermore, we investigated the phosphorylation of necroptotic RIPK1, RIPK3 and MLKL. No phosphorylation was detected, but a 33-kDa fragment of RIPK1, corresponding to inhibitory caspase-8-mediated digestion [6], was observed upon STING agonist treatment (Fig. 4B).

Because we previously demonstrated that STING agonist-induced monocyte RCD and IFNα secretion were both inhibited by the TBK1 inhibitor (TBK1i) [19], we examined the effect of TBKi on STING agonist-dependent caspase activation (Fig. 4C). TBKi nearly eliminated the STING agonist-induced activation of the analyzed caspases (Fig. 4C), including those potentially cross-reacting with the caspase-1 assay [31] (Fig. S5C). Remarkably, in untreated monocytes, TBK1i alone increased basal caspase activity (Fig. S5B). This corresponds with our previous results, where TBKi mildly depleted monocytes [19], and with reported TBK1-mediated inhibition of NLRP3 inflammasome activity [34]. However, upon STING agonist treatment, no further activation occurred, confirming that TBK1i blocked STING agonist-dependent caspase activation (Fig. 4C).

Additionally, to assess the immunogenic and inflammatory potential of STING agonist-induced monocyte RCD, we analyzed the release of DAMPs—extracellular ATP (eATP) and the high-mobility group box 1 protein (HMGB1), both markers of immunogenic cell death [1]. However, whereas the pyroptosis activator nigericin triggered eATP secretion, STING agonists induced neither eATP nor HMGB1 release (Fig. S5D).

### STING agonists induce mitochondrial dysfunction in monocytes

**Fig. 5:**
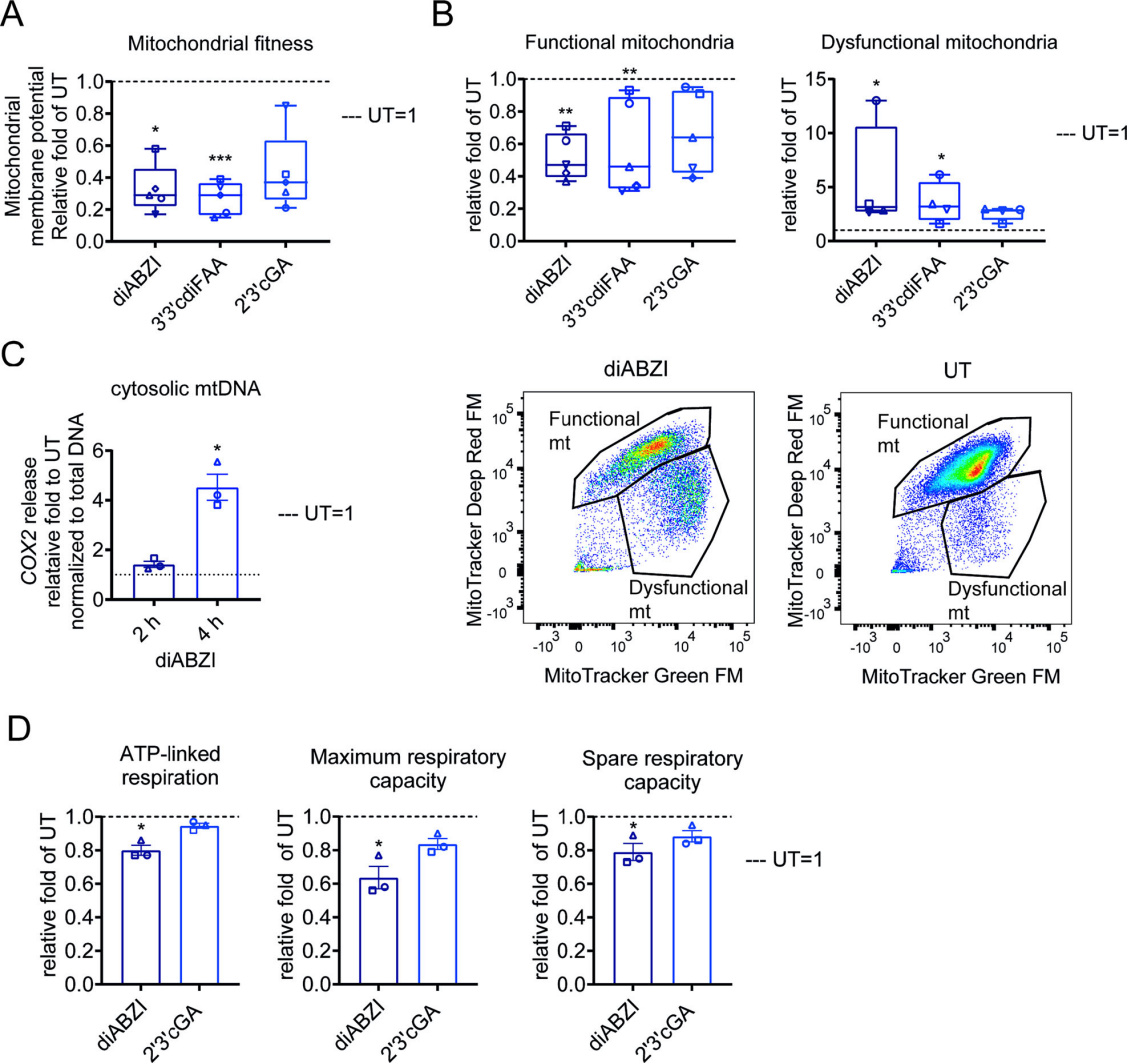
STING agonists induce mitochondrial dysfunction and reduce oxidative respiration in monocytes. **A** The effect of STING agonists on mitochondrial fitness was determined as the change in median fluorescence intensity (MFI) of MitoSpy Orange CMTMRos staining in the myeloid population, using multiparametric flow cytometry-based analysis. PBMCs (*n* = 5 donors) were treated for 4 h with STING agonists: diaminobenzimidazole-based agonist (diABZI), 3′,3′c-di(2′F,2′d-AMP) (3′3′cdiFAA) and 2′,3′-cGAMP (2′3′cGA). Untreated cells (UT) were used as control samples. The data are presented as the relative fold difference in MFI between treated samples and UT (UT = 1, dashed line). Individual donors are represented by different symbols. Data were analyzed with the Friedman test with the uncorrected Dunn’s test for multiple comparisons. *P < 0.05, **P < 0.01, ***P < 0.001. **B** The effect of STING agonists on mitochondrial function of enriched monocytes was determined using MitoTracker Green FM and Deep Red FM staining. Enriched monocytes (*n* = 5 PBMC donors) were treated for 4 h with the diaminobenzimidazole-based agonist (diABZI), 3′,3′c-di(2′F,2′d-AMP) (3′3′cdiFAA) and 2′,3′-cGAMP (2′3′cGA). Untreated cells (UT) were used as control samples. The effect of STING agonists on the mitochondrial function of enriched monocytes is presented as the relative fold difference in the frequency of monocytes with functional/dysfunctional mitochondria between treated samples and the baseline of UT (UT = 1, dashed line). Individual donors are represented by different symbols. The data were analyzed with the Friedman test with uncorrected the Dunn’s test for multiple comparisons. *P < 0.05, **P < 0.01. Representative example of enrichment of monocytes with dysfunctional mitochondria (mt). **C** STING agonists induce the release of mitochondrial DNA (mtDNA) into the cytoplasm analyzed by qPCR of *COX2* locus in the cytosolic fraction, presented as relative fold to cytosolic mtDNA in untreated cells (UT = 1, dashed line), normalized to total cellular *COX2* (*n* = 3 PBMC donors). Due to the sample requirements of the method and the maximum yield of monocyte enrichment, the treatment was limited to the diaminobenzimidazole-based agonist (diABZI). Bar graphs represent mean ± SEM. Individual donors are represented by different symbols. The data were analyzed with the Paired *t*-test. *P < 0.05. **D** The effect of STING agonists on oxidative respiration of enriched monocytes was analyzed by an Oxygraph 2K respirometer (*n* = 3 PBMC donors). Because of the sample requirements of the method and the maximum yield of monocyte enrichment, the treatment was limited to the diaminobenzimidazole-based agonist (diABZI) and 2′,3′-cGAMP (2′3′cGA). Untreated cells (UT) were used as control samples. Monocytes were treated for 2 h, which was the time point when the mitochondrial dysfunction could already be detected (Fig. S7B). The data are presented as the relative fold difference in the respective parameters between treated samples and UT (UT = 1, dashed lines). Bar graphs represent mean ± SEM. Individual donors are represented by different symbols. The data were analyzed with the Friedman test with the uncorrected Dunn’s test for multiple comparisons. *P < 0.05.

As RCD can be initiated extrinsically via membrane receptors or intrinsically through mitochondria [1], we next analyzed the effect of STING agonists on mitochondrial fitness and function. Upon 4 h of treatment, STING agonists lowered the median fluorescence intensity (MFI) of mitochondrial membrane potential-based staining (MitoSpy Orange CMTRos dye) in the PBMC myeloid population (‘lin− myelo’, Fig. S6), suggesting reduced mitochondrial fitness (Fig. 5A). We further verified the results in enriched monocytes using another mitochondrial membrane potential-sensitive dye, tetramethylrhodamine methyl ester (TMRM), once again showing a decrease of MFI upon 4-h STING agonist treatment (Fig. S7A). Moreover, STING agonists induced mitochondrial dysfunction in enriched monocytes already upon 2 h of treatment (Fig. S7B), with a pronounced effect upon 4 h (Fig. 5B). Furthermore, we demonstrated the release of mtDNA, detected as the cytochrome c oxidase subunit 2 (*COX2*) gene, into the cytoplasm upon 4 h STING agonist treatment (Fig. 5C), suggesting mitochondrial damage, likely accompanying the activation of the intrinsic apoptotic pathway.

Having proved the involvement of mitochondria in STING agonist-induced monocyte RCD, we investigated the effect of STING agonists on the metabolism of enriched monocytes using respirometry analyses. The STING agonists reduced mitochondrial ATP-linked respiration, maximum respiratory capacity and spare respiratory capacity when compared with UT, although only diABZI significantly (Fig. 5D). We further explored the metabolism using a Seahorse Extracellular Flux Analyzer. The STING agonist treatment led to a decreasing oxygen consumption rate (OCR) starting around 80 min, corresponding to an increasing extracellular acidification rate (ECAR) starting around 50 min upon treatment of enriched monocytes, suggesting a switch from oxidative respiration to glycolysis (Fig. S7C).

## Discussion

This report builds on our previous findings that a large portfolio of both synthetic and natural STING agonists induces the secretion of interferons and proinflammatory cytokines in PBMCs [17, 19], along with rapid monocyte cell death, which is inseparable from the type I IFN secretion [19]. As monocytes are responsible for innate immune surveillance against invading pathogens [27], this process may contribute to the regulation of cytokine secretion and subsequent immune responses important in antiviral and antitumoural defenses [35–37]. To determine whether monocyte death is unique to the cGAS–STING pathway, we compared the effects of three distinct STING agonists (lipophilic, synthetic, diaminobenzimidazole-based agonist (diABZI); polar, synthetic CDN-based STING agonist 3′,3′c-di(2′F,2′d-AMP) (3′3′cdiFAA); and polar, natural eukaryotic CDN-based STING agonist 2′,3′-cGAMP (2′3′cGA)) on induced cytokine signatures and frequencies of PBMC immune subsets with those of multiple TLR agonists. Unlike TLR agonists, only STING agonists stimulated the secretion of a unique cytokine signature, combining type I and III IFNs, proinflammatory cytokines (TNFα, IL1β, IL18, IL6) and IFNγ, produced secondarily upon STING agonist treatment, likely due to immune subset crosstalk [18, 19]. We previously demonstrated that this cytokine profile had stronger anti-HBV effects than those induced by the TLR7 agonist [18]. Whereas the TLR7/8 agonist-induced cytokine profile also exhibited stronger anti-HBV properties compared to single-acting TLR activators [28], it primarily stimulated proinflammatory cytokines with limited amounts of type I and III IFNs. Because these interferons are key players in anti-HBV immunity [38], we propose that STING agonists may generate a more complex cytokine signature, potentially leading to more effective outcomes in anti-HBV therapy compared to TLR activators.

Overall, TLR agonists in our study did not significantly affect cMo frequencies, although there was considerable donor-to-donor variability. iMo frequencies were the most sensitive to all treatments, likely due to their role in cytokine secretion, T cell activation and antigen presentation [27, 39]. The depletion of both iMo and nMo could be mediated by the previously described CD16 shedding [40]. Conversely, no enrichment of monocyte or DC subsets was observed following TLR treatment, except for pDC enrichment upon TLR9 activation, likely linked to type I and III IFN secretion [29]. Research on dual-acting TLR agonist and their effects on monocytes remains limited. Studies testing potential vaccine adjuvants in non-human primates have shown that the TLR7/8 agonist Resiquimod induces nMo expansion [41], whereas research on the effects of dibutyl phthalate in whole human blood has reported no changes in CD16 mean fluorescent intensity in monocytes upon Resiquimod treatment [42]. However, this analysis is not directly comparable to our setting, as monocyte subpopulations were not analyzed in the study. To the best of our knowledge, the effect of TLR2/7 agonist Adilipoline on monocytes remains unexplored.

Strikingly, only STING agonists caused a near-complete depletion of all three monocyte subsets, accompanied by the loss of CD16 and CD14. The CD16 depletion may be attributed to previously reported CD16 shedding [40], whereas CD14 was likely degraded, as we previously found no evidence of CD14 shedding upon STING agonist treatment [19]. Importantly, the loss of monocyte markers and the immunophenotypic enrichment of DC-like subsets were not caused by monocyte differentiation, because the enriched population displayed an apoptotic phenotype.

Furthermore, none of the PRR activators significantly affected NK and B cell frequencies, whereas T cells were mildly enriched upon TLR7/8 and TLR2/7 agonist treatment, likely due to induced proliferation [43, 44]. The TLR9 agonist treatment increased NKT cell frequencies, possibly via pDC-mediated induction [45]. The STING agonist treatment did not affect the analyzed lymphoid subset frequencies, and, contrary to previous findings [19], no NK cell depletion was observed, possibly reflecting donor-to-donor variability. Nevertheless, the effects of STING agonists on NK cells merit further investigation.

As we have verified, the STING agonists uniquely affected PBMCs by promoting the secretion of a broad cytokine portfolio and inducing monocyte depletion. To better understand the STING agonist-induced response, including immunogenic cell death [35, 37, 46], we thoroughly investigated the mechanism of monocyte RCD. Previously, we described that monocytes undergoing depletion exhibited apoptotic characteristics; however, the secreted IL1β suggested a possible involvement of pyroptosis [19]. Therefore, we investigated the three most prominent RCDs pathways implicated in cGAS–STING signaling—apoptosis, pyroptosis and necroptosis [26]. Interestingly, STING agonists can also induce a combination of these RCDs—PANoptosis [24, 25]. We have demonstrated that STING agonists trigger both apoptosis and pyroptosis in monocytes, as evidenced by the activation of apoptotic caspases (-3, -7, -9 and -8) and pyroptotic markers (active caspase-1, cleaved GSDMD, and secreted IL1β and IL18, both of which require processing by the caspase-1 inflammasome [1]), respectively. The detection of active caspases and cleaved GSDMD in enriched monocytes indicated that RCD was induced directly, independently of cell-to-cell crosstalk. Moreover, GSDMD was cleaved by both caspase-1 and caspase-3, where caspase-3-mediated cleavage inhibits GSDMD pore-forming activity [47, 48], suggesting a possible regulatory balance between apoptosis and pyroptosis that may modulate cytokine secretion and RCD in the context of cGAS–STING signaling. Moreover, both pyroptotic [49–51] and apoptotic [52–54] mechanisms can suppress cGAS–STING pathway-mediated type I IFN secretion. Additionally, caspase-8 may also activate GSDMD and promote the maturation of IL1β and IL18, as observed in the context of bacterial infections [2, 33, 55–57].

We then investigated the role of necroptosis, as the STING agonist diABZI was previously shown to induce PANoptosis in murine bronchoalveolar immune cells and trigger the DNA-mediated acute respiratory distress syndrome in experimental animals, suggesting that STING agonists may not be suitable for treating respiratory infections [24]. Furthermore, genomic instability induced cGAS–STING-mediated PANoptosis in diffuse large B-cell lymphoma cell lines expressing STING, highlighting the therapeutic potential of STING agonists in STING-expressing cancer cells [25]. However, in our study, STING agonists did not activate the necroptotic pathway, as we detected no phosphorylation of RIPK1, RIPK3 or MLKL. Instead, we observed a RIPK1 fragment corresponding to caspase-8-mediated cleavage, which inhibits the necroptotic cascade [2, 6, 58]. Therefore, we concluded that PANoptosis was not activated by STING agonists in human monocytes.

Interestingly, although STING agonists-induced RCD triggered the secretion of IL1β and IL18, DAMPs (eATP and HMGB1) were not released, indicating that STING agonist-induced monocyte RCD may be less inflammatory than nigericin-induced pyroptosis. This corresponds with our hypothesis that cGAS–STING signaling orchestrates a regulated interplay between apoptotic and pyroptotic mechanisms.

To demonstrate the association between caspase activation and the cGAS–STING pathway, we inhibited TBK1 [26] using the TBKi MRT68601 hydrochloride, which previously fully blocked TBK1-mediated IRF3-dependent type I IFN production while simultaneously preventing monocyte depletion [19]. Notably, NFκB-dependent proinflammatory cytokine secretion was maintained or only partially inhibited [19], as NFκB can also be activated by IKKε independently of TBK1 [59]. This also excluded the involvement of NFκB in STING agonist-induced monocyte RCD. Indeed, STING agonist-mediated activation of all the analyzed caspases was blocked by TBK1i, suggesting direct cGAS–STING-dependent caspase activation. Additionally, simultaneous caspase-9, -8 and -3/7 inhibition corresponds to their connection in apoptosis [1]. However, it remains unclear whether caspase-1 was also activated by caspase-8, as observed in bacterial infections [2, 60], by active caspases-3/7, which were previously shown to activate NLRP3-dependent inflammation upon TLR signaling [55, 57], or independent activation of the NLRP3 inflammasome [26, 61].

Although caspase-8 acts as an initiator caspase in extrinsic apoptosis, it can also trigger apoptosis intrinsically and participate in pyroptosis or PANoptosis [2, 7, 8, 33, 62]. Since the STING agonist-mediated activation of caspase-9 confirms the involvement of the intrinsic apoptotic pathway, we presume that, in our setting, caspase-8 may be activated downstream of proapoptotic proteins BAX and BAK acting on mitochondria [55, 57]. Importantly, BAX has been shown to interact with phosphorylated IRF3 upon STING activation, suggesting the BAX-IRF3 axis of the intrinsic apoptosis stimulation [21]. Recently, Chauhan et al. [63]. described that murine bone marrow-derived macrophages underwent apoptosis during the late stages of *Coxiella burnetii* infection. This RCD was dependent on STING and involved BAX–IRF3-mediated activation of intrinsic apoptosis characterized by the release of cytochrome-c (cyt-c) and the activation of caspase-9 and -3, as well as caspase-8. The authors also observed mitochondrial depolarization, accompanied by the release of mitochondrial DNA (mtDNA) into the cytosol, which further amplified cGAS–STING signaling [63]. Because STING agonist-mediated induction of IRF3-dependent type I IFN secretion was inseparable from monocyte death in our previous study [19], and inhibition of the IRF3 axis by TBK1i blocked the activation of all the analyzed caspases, we investigated the mitochondrial state in human monocytes upon STING agonist treatment. In line with already published results [64], STING agonist treatment reduced mitochondrial fitness monitored as a decrease in the mitochondrial membrane potential, induced mitochondrial dysfunction, and suppressed mitochondrial oxidative respiration. The reduction in mitochondrial respiration was accompanied by increased glycolysis, an already reported metabolic shift [64]. The switch from oxidative respiration to glycolysis may be connected to the induced proinflammatory cytokine secretion [65]. Moreover, in line with activation of the intrinsic apoptotic pathway, we also observed increased amounts of mtDNA upon STING agonist treatment. Although mitochondrial damage and mtDNA were reported as upstream inducers of the cGAS-STING pathway in mouse models of obesity-related diabetes [66, 67], these observations do not contradict our results and reported downstream mtDNA release in *Coxiella burnetii* infection acting as positive feedback for cGAS-STING signaling amplification [63]. We overall demonstrate that STING agonists affected mitochondrial function, leading to mitochondrial damage accompanied by caspase-9 activation and mtDNA release into the cytoplasm. Thus, we also assume the release of cyt-c [63]. These aspects will be further investigated to elucidate their role in the mechanisms of STING-driven RCD.

**Fig. 6:**
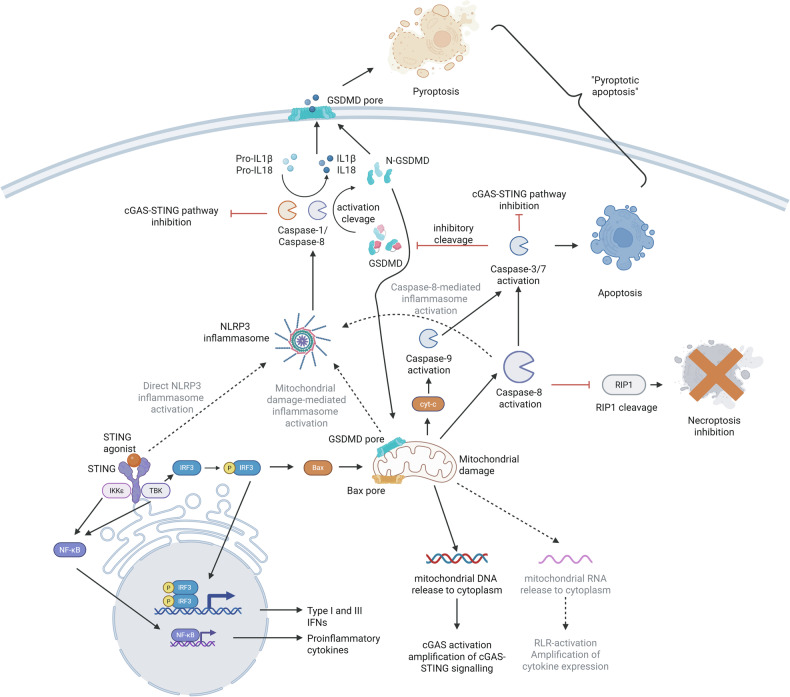
Scheme of STING agonist-induced monocyte cell death mechanism of ‘pyroptotic apoptosis’ based on our results and evidence from the literature. Synthetic or natural STING agonists bind the STING protein and induce its oligomerization, which allows for the activation of TANK-binding kinase 1 (TBK1) and IκB kinase ε (IKKε) [59]. The kinases then trigger the nuclear translocation of the transcription factors interferon regulatory factor 3 (IRF3) and nuclear factor κB (NFκB) and the subsequent secretion of type I and III interferons (IFNs) and proinflammatory cytokines. Phosphorylated IRF3 can also interact with the proapoptotic protein Bax [21] and induce the intrinsic apoptotic pathway combined with cytochrome-c (cyt-c) [63] release and caspase-9 activation. This process also activates caspase-8, which inhibits the necroptotic pathway via RIPK1 cleavage and, together with caspase-9, triggers effector apoptotic caspases-3/7. Moreover, this process is accompanied by mitochondrial damage and the release of mitochondrial DNA into the cytoplasm. Released DNA can further amplify cGAS–STING pathway signaling [63, 66, 67]. Additionally, mitochondrial RNA could also escape and activate the RIG-I-like receptor pathways, resulting in amplified cytokine secretion[69]. STING agonists also activate pyroptotic caspase-1 and gasdermin-D (GSDMD) and release mature interleukin-1β (IL1β) and IL18, probably via the NLRP3 inflammasome, which can be induced by mitochondrial damage, by caspase-8 or directly by activation of the cGAS–STING pathway [1]. GSDMD forms pores both on the plasma membrane and on the mitochondrial membrane, probably further amplifying the mitochondrial damage and subsequent signaling. On the other hand, caspases-3/7 cleave and inhibit GSDMD, therefore limiting the pyroptotic pathway and mitochondrial damage, while both caspases-3/7 and caspase-1 inhibit the cGAS–STING pathway itself. Overall, the described STING agonist-induced ‘pyroptotic apoptosis’ of monocytes could present a complex mechanism for the precise regulation of cytokine secretion and activation of subsequent adaptive immune processes. The figure was created in BioRender. Pimkova Polidarova, M. (2025) https://BioRender.com/0jmybvp.

Because the reduction in mitochondrial function and oxidative respiration preceded the activation of caspases in human monocytes, we hypothesized that STING agonist-induced RCD could be initiated via the STING–IRF3–BAX pathway [21, 63] (Fig. 6). BAX-mediated mitochondrial membrane pore formation may then trigger the apoptotic pathway, accompanied by cyt-c (not shown in our study) and mtDNA release, leading to the activation of apoptotic caspases-9, -3/7 and -8 and pyroptotic caspase-1 [2, 56, 63]. Caspase-8 simultaneously inhibits necroptosis by cleaving RIPK1 [6]. Caspase-1, and possibly caspase-8, activate the inflammasome, allowing for the maturation and secretion of IL1β and IL18 via GSDMD pores [1, 56]. GSDMD pores have also been shown to target the mitochondrial membrane, further increasing mitochondrial damage via outer and inner mitochondrial membrane permeabilization [68]. Both BAX- and GSDMD-mediated mitochondrial perforation could facilitate the release of mtDNA into the cytoplasm, further enhancing cGAS–STING pathway activation [63]. Additionally, mtRNA escape could trigger RIG-I-like receptor pathways, acting synergistically with cGAS–STING-mediated cytokine secretion [69].

However, this induced RCD appears to be specific for monocytes, as observed after 4 h of treatment, whereas previous studies reported T cell death only after 24-h or longer STING agonist treatment [22, 70]. However, these studies primarily focused on enriched murine splenic T cells; therefore, they lacked the context of other immune cells, which could provide additional pro-survival signals [22, 70]. Moreover, T cells were not depleted but produced IFNα and expressed the CD69 activation marker upon 18 h of STING agonist treatment [18]. Other studies have also reported no toxicity of STING agonists on human T cells [64, 71]. STING agonist-induced RCD is likely species-specific, as our findings in human monocytes do not correspond with the PANoptosis observed in murine bronchoalveolar immune cells [24].

To summarize, we have identified that STING agonists induce RCD in human monocytes, integrating both apoptotic and pyroptotic processes. This ‘apoptotic pyroptosis’ [2] involving mitochondrial dysfunction (likely driven by the STING–IRF3–BAX pathway) [63], caspase-9 and caspase-8 activation may serve as a regulatory mechanism for STING agonist-stimulated cytokine secretion. ‘Apoptotic pyroptosis’ could function as a negative regulatory feedback loop, as both apoptotic and pyroptotic proteins can inhibit the cGAS–STING pathway [49–54]. Although proinflammatory conditions are important for initiating acute immune responses, prolonged exposure to proinflammatory cytokines can lead to a cytokine storm, a life-threatening condition [72]. Moreover, the resolution of primary cytokine secretion is needed for subsequent adaptive immune responses and effective immune defense [73]. Thus, rapid monocyte RCD may provide a mechanism to efficiently halt cytokine production, allowing for subsequent immune activation, whereas monocytes can be quickly replenished from the bone marrow [74]. Conversely, in vivo, this process may occur locally, affecting only a small population of cells. Overall, our findings provide new insights into the outcomes of cGAS–STING signaling in human monocytes, contributing to the understanding of innate immune processes and the development of STING agonist-based therapeutic strategies.

## Materials and methods

### Cell cultivation and treatment

All cells were cultivated in PBMC medium (RPMI 1640, ThermoFisher Scientific, Waltham, Massachusetts, USA), 10% fetal bovine serum (ThermoFisher Scientific) at 37 C and 5% CO_2_ atmosphere (referred to as standard conditions), unless stated otherwise.

The activators of PRR pathways were purchased from Invivogen (San Diego, California, USA) and used as previously determined [18, 19, 28]: 50 nM TLR7 agonist (GS-9620), 25 µg/ml TLR3 agonist (poly I:C), 1 µM TLR9 agonist (CpG-A, ODN2216), 4 µg/ml TLR7/8 agonist (Resiquimod), 5 µg/ml TLR2/7 agonist (Adilipoline), 1 µM STING agonist diABZI, 30 µM STING agonist 3′,3′c-di(2′F,2′d-AMP), and 100 µM STING agonists 2′,3′-cGAMP. The three STING agonists differ in their 50% effective concentration (EC_50_); hence, we compensated for this difference by choosing predetermined concentrations above EC_50_, which effectively triggered the cGAS–STING pathway [18, 19]. Cell death activators were used as positive controls in concentrations recommended by the manufacturers: 2 µM staurosporine (Merck, Rahway, New Jersey, USA) and 20 µM nigericin (Bio-Rad, Hercules, California, USA) for the induction of apoptosis and pyroptosis, respectively. Pretreatment for 30 min with 5 µM TBK1 kinase inhibitor MRT 68601 hydrochloride (Tocris Bioscience, Bristol, UK) was used as previously determined [19]. Untreated cells were used as a negative control.

For cytokine and immunophenotype determination, 3 × 10^6^ cells/ml 100 µl were seeded in a 96-well format and incubated with tested compounds in standard conditions for 16 h. [18, 19] For cell death pathway analyses, cells were incubated for 4 h [19]. For flow cytometry-based caspase assays, the cells were cultivated at 1 × 10^6^ cells/ml in 300 µl in a 48-well format and for the reporter-based caspase assays at 1.5 × 10^6^ cells/ml in 100 µl in a 96-well format. To study mitochondrial fitness and function, cells were seeded at 3 × 10^6^ cells/ml in 100 µl in a 96-well format and incubated for 2 and 4 h. For immunodetection analyses of cell death cascade proteins using western blotting, cells were cultivated for 4 h at 15 × 10^6^/ml in 1 ml in a 24-well format. For analyses of mtDNA release into cytosol, cells were cultivated for 4 h at 3.3 × 10^6^/ml in 2 ml in a 24-well format. For oxygen consumption analyses, a total of 15 × 10^6^ cells were treated for 2 h at 3 × 10^6^ cells/ml in 2 ml in a 12-well format. For Seahorse analyses, 1.5 × 10^6^ cells/ml were treated in 100 µl for 2 h prior to measurement, or for 218 min continuous monitoring. Further details are presented below in the section Seahorse analyses. All seeding conditions were optimized for the respective analyses.

### Analyses of PBMCs or enriched monocytes

Buffy coats from healthy individuals were obtained from the Institute of Hematology and Blood Transfusion (IHBT, Prague, Czech Republic). Informed written consent was obtained from each individual enrolled. The study was approved by the institutional review board of the IHBT (evidence number 10/10/2022) and was done in accordance with the Declaration of Helsinki, along with good clinical practice guidelines.

PBMCs were isolated as previously described [18, 19] from fresh buffy coats of healthy individuals. Alternatively, monocytes were obtained using the RosetteSep Human Monocyte Enrichment cocktail (Stemcell, Vancouver, Canada) according to the manufacturer’s instructions. Monocyte purity was verified by flow cytometry-based immunophenotyping (Fig. S8) using the gating strategy described below (Fig. S2, Table S1). The cells were treated in a 1:1 ratio of tested compounds diluted in PBMC medium.

The cells were collected for subsequent analyses (see below), and the culture medium was collected for cytokine profiling. The concentration of cytokines (IFNα, IFNβ, IFNω, IL29 (IFNλ1), IFNγ, TNFα, IL6, IL1β and IL18) was determined by performing ProcartaPlex assays (Thermo Fisher Scientific) using a MAGPIX® System (DiaSorin, Saluggia, Italy) or flow cytometry-based readout (unpublished results).

### Reporter-based analyses of cell death pathways

Active caspases were detected in enriched monocytes using the Caspase-Glo 3/7 Assay System, Caspase-Glo 8 Assay System, Caspase-Glo 9 Assay System and Caspase-Glo 1 Inflammasome Assay (Promega, Madison, Wisconsin, USA) according to the manufacturer’s protocol. Luminescence signal was detected at the time point of the greatest background-to-noise difference, predetermined as follows: 30 min for caspase-3/7 and 60 min for caspase-1, -9 and -8, respectively, using a Spark spectrofluorometer (Tecan, Männedorf, Switzerland).

### Flow cytometry-based detection of active caspases

Active caspases were detected in PBMCs using FLICA kits labeled with the FAM fluorochrome (FAM-FLICA Caspase-3/7 and Pyroptosis FAM Caspase-1 kits, BioRad; CaspaTag Caspase-8 In Situ Assay Kit, Merck) according to the manufacturer’s instructions. The 30-min caspase staining was followed by dead cell staining and immunophenotyping (see below).

### Immunophenotyping

The PBMCs were immunophenotyped by a previously established protocol [18, 19], using the antibodies listed in Table S1 (General immunophenotyping) and Table S2 (Flow cytometry-based detection of active caspases). Control samples of unstained cells, positive control samples for dead cells (PBMCs in phosphate-buffered saline (PBS), Merck, incubated for 5 min at 65 °C), fluorescence-minus-one and isotype controls (where relevant) were also prepared. Immunophenotyping was assessed using a BD LSRFortessa flow cytometer (BD Biosciences, Franklin Lakes, New Jersey, USA), and data were acquired using the software FACS Diva (version 8.0.1, BD Biosciences). Debris was excluded by forward and side scatter gating, followed by doublet exclusion. For general immunophenotyping, dead cells were excluded, and the populations of interest were gated as follows: CD3+CD19−CD56− T cells, CD3+CD19−CD56+ NKT cells, CD3−CD19+CD56- B cells, CD3−CD19−CD56+ NK cells. To exclude triple-negative lymphoid cells (CD3−CD56−CD19−), the granular population was then gated based on forward and side scatter parameters (‘lin- myeloid’ cells), followed by further analyses of the myeloid subsets: CD3−CD19−CD56−CD14+CD16− cMo, CD3−CD19−CD56−CD14+CD16+ iMo, CD3−CD19−CD56−CD14−CD16+ nMo, CD3−CD19−CD56−CD14−CD16−CD123+CD11c- pDC-like cells, CD3−CD19−CD56−CD14−CD16−CD123+CD11c+ DC-like cells, and CD3−CD19−CD56−CD14−CD16−CD123−CD11c+ cDC-like cells (Fig. S2). The data were analyzed using FlowJo software (version 10, BD Biosciences) [75].

Changes in the phenotype of lin- myeloid cells upon STING agonist treatment were also analyzed using unbiased multiparametric analyses. First, the quality control plugin PeacoQC was employed to exclude ‘Bad Events’ from the analysis. The gating strategy up to the ‘lin- myeloid’ subset was applied manually, and the gates were concatenated across the samples. The samples were further analyzed using the dimensionality reduction plugin UMAP and the plugins FlowSOM for automatic clustering and Cluster Explorer for cluster phenotype determination in FlowJo software (version 10, BD Biosciences) [75].

For the analysis of caspase activation, debris and doublets were excluded as described above, and the populations of interest were gated as described above up to the monocyte populations. FAM-FLICA and live/dead marker positivity were determined in the ‘lin- myeloid’ cell population (Fig. S4).

### Flow cytometry-based detection of mitochondrial fitness based on mitochondrial membrane potential

Mitochondrial fitness was analyzed in PBMCs using 50 nM MitoSpy Orange CMTMRos (BioLegend, San Diego, California, USA) added upon 4 h of treatment according to the manufacturer’s protocol. The cells were then collected for immunophenotyping as previously described [17–19], followed by live/dead marker and surface antibody staining described in Table S3 and analyses using the gating strategy described in Fig. S6.

### Flow cytometry-based detection of mitochondrial membrane potential

Mitochondrial potential changes obtained with MitoSpy Orange CMTMRos (BioLegend) were verified in enriched monocytes using MitoProbe TMRM Kit for Flow Cytometry (ThermoFisher Scientific, Waltham, Massachusetts, USA). Enriched monocytes were stained for 30 min at 37 °C with TMRM and live/dead marker (Table S4) upon 2 and 4 h of according to the manufacturer’s protocol and analyzed using the gating strategy described in Fig. S9.

### Flow cytometry-based detection of mitochondrial dysfunction

Enriched monocytes were incubated for 2 or 4 h with STING agonists and then stained with 5000× diluted Mitotracker Green FM (ThermoFisher Scientific), 5000× diluted Mitotracker Deep Red FM (ThermoFisher Scientific) [76], and 250× diluted Zombie NIR viability dye in PBS for 30 min. The cells were washed once with PBS, followed by surface antibody staining as described in our previous studies [17–19] using the antibodies and gating strategy listed in Table S5 and Fig. S10.

### Immunodetection of regulated cell death in enriched monocytes upon cGAS–STING pathway activation

The activation of regulated cell death mechanisms upon cGAS–STING pathway activation in enriched monocytes was monitored by the immunodetection of pro-caspases -3, -7, -8, and -1 and their respective cleaved forms, GSDMD and its cleaved forms, and RIP1, RIP3 and MLKL kinases and their phosphorylated or cleaved forms (the antibodies are listed in Table S5). Control untreated cells (UT) and cells incubated for 4 h with diABZI were lysed in 100 µl of ice-cold RIPA buffer (50 mM tris–HCl (VWR, Radnor, Pennsylvania, USA), 1% Nonidet P-40 (Merck), 150 mM NaCl (Merck), 0.5% sodium deoxycholate (ThermoFisher Scientific), 0.1% sodium dodecyl sulfate (SDS, Merck)) containing inhibitors of proteases (cOmplete™, EDTA-free Protease Inhibitor Cocktail, Roche (Basil, Switzerland)) and phosphatases (PhosSTOP™, Merck). The lysates were then treated with benzonase nuclease (Merck) for 10 min at RT. The samples were mixed with a sample loading buffer (0.3 M tris–HCl (VWR) pH 6.8, 5% SDS (Merck), 50% glycerol (Penta, Prague, Czechia), 2.5% β-mercaptoethanol (VWR), 0.05% bromophenol blue (Cayman Chemical, Ann Arbor, Michigan, USA)), incubated for 5 min at 95 °C. The proteins were separated using SDS polyacrylamide gel electrophoresis the tris–glycine SDS buffer system [77] in a mini vertical electrophoresis unit (Mini-PROTEAN Tetra Vertical electrophoresis Cell, Bio-Rad). Gels were blotted onto a polyvinylidene difluoride (PVDF) membrane (Amersham Hybond 0.45 µm; GE Healthcare, Chicago, Illinois, USA) in a blotting buffer (25 mM Tris (VWR), 192 mM glycine (VWR), 20% methanol (Penta)) using a Mini Trans-Blot system (Bio-Rad) for 1 h at 100 V, 4 °C. Subsequently, the membrane was blocked with 2.5% bovine serum albumin (Merck) in TBS-T (tris-buffered saline pH 7.4—25 mM tris (Merck), 150 mM NaCl (Merck), 2 mM KCl (Penta) containing 0.05% Tween-20 (VWR)) and incubated with the primary antibody in blocking buffer for 2 h at RT and then with the secondary antibody in blocking buffer for 1 h at RT (the list of antibodies and respective dilutions and buffers is presented in Table S6). The proteins were visualized using an Odyssey CLx Imager (Li-Cor Biosciences, Lincoln, Nebraska, USA).

### Quantification of mtDNA release into the cytoplasm

The release of mtDNA upon 4 h of diABZI treatment was analyzed as previously reported [78] using NucleoSpin Gel and PCR Clean-up (Macherey-Nagel, Düren, Germany) for isolation of the cytosolic fraction and NucleoSpin DNS Tissue (Macherey-Nagel) for isolation of total DNA. Due to the high sample requirement, only diABZI was selected as a representative STING agonist. Mitochondrial COX2 gene was quantified by qPCR in both total DNA and cytosolic DNA fraction using Luna Universal qPCR Master Mix (New England Biolabs, Ipswich, Massachusetts, USA) and LightCycler 480 II instrument (Roche, Basel, Switzerland) using primers of following sequences: forward: 5’-AATCGAGTAGTACTCCCGATTG-3’, reverse: 5’-TTCTAGGACGATGGGCATGAAA-3’ [78] (Eurofins, Luxembourg, Luxembourg).

### Respirometry analyses

The high-resolution Oxygraph 2k instrument (Oroboros, Innsbruck, Austria) was used to determine the consumption of oxygen at 2 h after treatment of enriched monocytes with STING agonists (diABZI and 2′,3′-cGAMP, as described above), following a protocol described in a previous study [79]. Untreated cells were used as a control sample. Briefly, the Oxygraph 2k device was calibrated in air, and the background was corrected with the cultivation medium. We loaded 7–10 × 10^6^ cells/ml in 2 ml of PBMC medium into the analysis chamber. Endogenous respiration was followed by treatment with 5 µM oligomycin A (ATP synthase inhibitor, Merck), 8–12 µM FCCP (carbonyl cyanide p-(trifluoromethoxy)-phenylhydrazone, an uncoupler of the electron transport chain and ATP synthase; the concentration required for full inhibition varied among individual donors, Merck), and 500–1000 µM KCN (a cytochrome oxidase inhibitor, Merck). Respiratory parameters were calculated as follows: ATP-linked respiration—ratio of endogenous (basal) oxygen consumption and oxygen consumption upon oligomycin A treatment, maximum respiratory capacity—ratio of oxygen consumption upon FCCP and oligomycin A treatment, spare respiratory capacity—ratio of oxygen consumption upon FCCP treatment and endogenous oxygen consumption.

### Seahorse analyses

Enriched monocytes were seeded onto Cell-Tac (Corning, New York, USA)-coated Seahorse XF HS Miniplates (Agilent Technologies, Santa Clara, California, USA) at 0.15 × 10^6^ cells/well in 50 µl of XF RPMI medium pH 7.4 (Agilent Technologies) supplemented with XF Glucose (Agilent Technologies) and XF Glutamine solutions (Agilent Technologies) according to the manufacturer’s instructions. For the monocytes to adhere to the Cell-Tac in a monolayer, the plates were spun for 1 min at 200 × *g* without brakes; 30 µl of medium was then added to reach the assay volume recommended by the manufacturer’s protocol. A Seahorse XF HS Mini Analyzer (Agilent Technologies) was used to monitor the effect of STING agonist treatment on monocyte respiration and glycolysis over time in an XF ATP-rate assay (Agilent Technologies) according to the manufacturer’s instructions. Because the monocytes were depleted as a result of the STING agonist treatment, normalization for the number of nuclei could not be applied at the end of the experiment (218 min).

### Statistical analyses

The GraphPad Prism 8 (GraphPad Software, Boston, Massachusetts, USA) was used to perform statistical analyses. Samples were excluded from the analyses if a technical error occurred during data acquisition. Remaining outliers were identified using ROUT method with Q = 1%. Data were normalized to untreated samples (UT) for each donor, where applicable. The number of biological replicates (*n*), i.e., individual donors, is reported in the figure legends. The experiments were performed in technical duplicates except for western blot and oxygraph analyses due to high sample requirements and a limited amount of biological sample. The Shapiro–Wilk normality test was used to determine the normal distribution of results. The Friedman test with the uncorrected Dunn’s test was used for multiple comparisons. Two-way ANOVA was used to compare the effect of caspase-1 inhibitor or TBK1i against non-inhibited conditions. Paired *t*-test was used to assess the mtDNA release into the cytosol. Statistical significance is indicated as either not significant at *P* > 0.5 or significant at *P < 0.05, **P < 0.01, ***P < 0.001, ****P < 0.0001.

## Supplementary information

SI

WB-supplementary

## Data Availability

The datasets generated and/or analyzed during the current study are available from the corresponding authors on reasonable request. Full western blot membranes represented by Fig. 4B and data of biological replicates are contained in the supplementary file WB-supplementary.pdf accompanying this manuscript.
