# Supplementary material for: STING agonists trigger monocyte death via apoptosis, pyroptosis, caspase-8 activation and mitochondrial dysfunction
Source: Cell Death Discov. 2025 Oct 31;11:494. doi: 10.1038/s41420-025-02786-1 (PMC12579220; doi:10.1038/s41420-025-02786-1)
Supplement: Supplementary file 1 — SI [file 41420_2025_2786_MOESM1_ESM.pdf]

Supporting information

STING Agonists Trigger Monocyte Death via Apoptosis, Pyroptosis, Caspase-8 Activation and Mitochondrial Dysfunction

Marketa Pimkova Polidarova[1,2], Lydie Plecita-Hlavata[3], Ivan Hirsch[1,2], Klara Grantz Saskova[1*], Andrea Brazdova[1*]

[1] Department of Genetics and Microbiology, Faculty of Science, Charles University, BIOCEV, Vestec, Czech Republic

[2] Institute of Organic Chemistry and Biochemistry of the Czech Academy of Sciences, Prague, Czech Republic

[3] Laboratory of Pancreatic Islet Research, Institute of Physiology of the Czech Academy of Sciences, Prague, Czech Republic

* Corresponding authors: andrea.brazdova@natur.cuni.cz (A.B.); saskova2@natur.cuni.cz(K.G.S.)

A

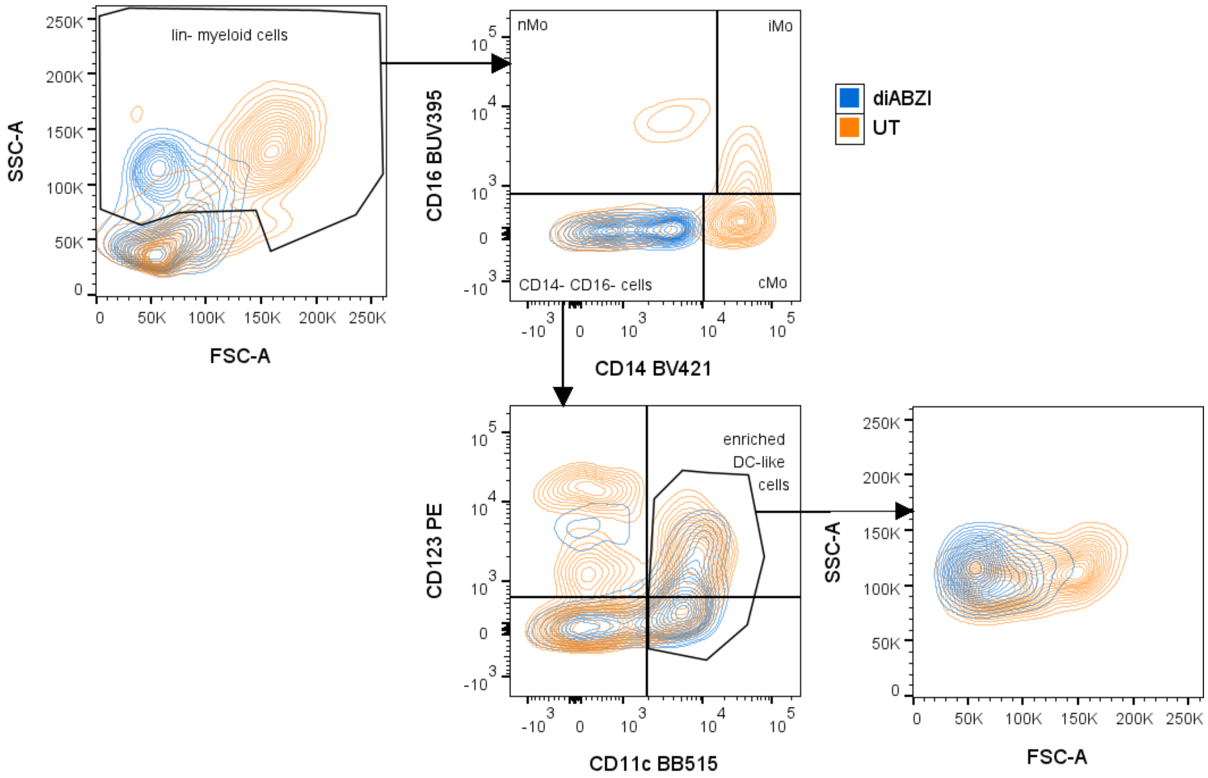
lin- myeloid cells
SSC-A
FSC-A
CD16 BUV395
CD14 BV421
nMo
iMo
cMo
CD14- CD16- cells
diABZI
UT
CD123 PE
CD11c BB515
enriched
DC-like
cells
SSC-A
FSC-A

B

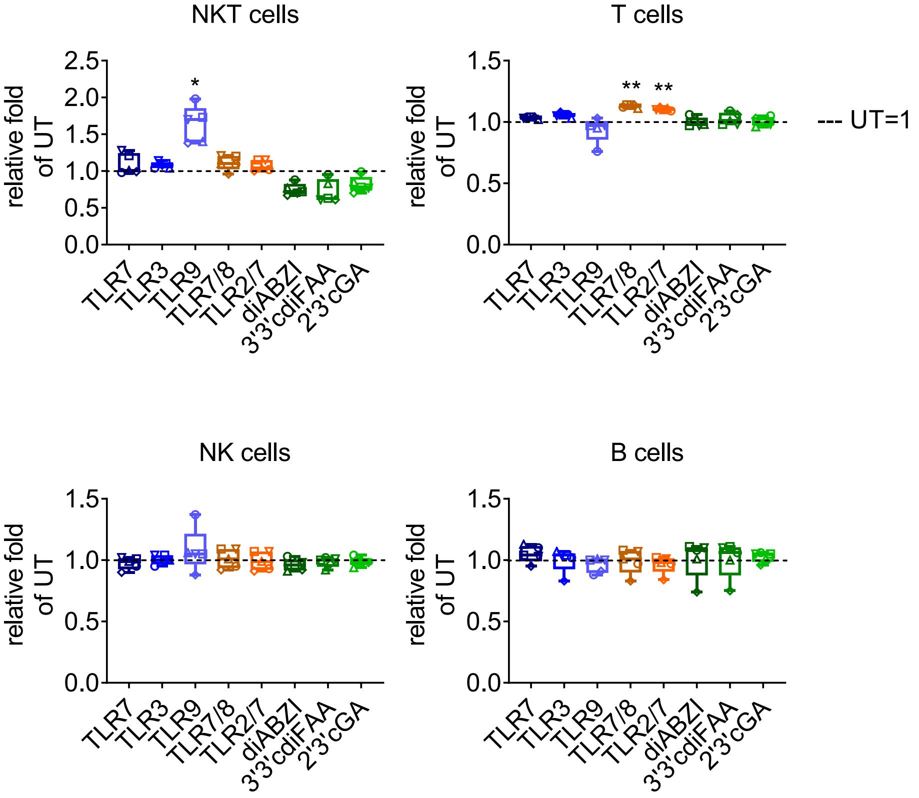
NKT cells
T cells
NK cells
B cells
relative fold
of UT
--- UT=1
TLR7
TLR3
TLR9
TLR7/8
TLR2/7
diABZI
3'3'cdiFAA
2'3'cGA

Figure S1: STING agonists induce the almost complete depletion of monocyte populations in PBMCs. Human PBMCs were treated for 16 h with TLR agonists: TLR7 agonist GS-9620, TLR3 agonist poly I:C (HMW), TLR9 agonist CpG-A (ODN2216), TLR7/8 agonist Resiquimod, TLR2/7 agonist Adilipoline, and the STING agonists: diaminobenzimidazole-based agonist (diABZI), 3′,3′c-di(2′F,2′d-AMP) (3′3′cdiFAA) and 2′,3′-cGAMP (2′3′cGA). Untreated cells (UT) were used as a control. **A** Representative example of changes in the myeloid population upon diABZI treatment based on the gating presented in Fig. S2. Upon diABZI treatment, monocytes loose CD14 and CD16 markers, enriching the DC-like population. However, the enriched DC-like population has a phenotype of dying cells, based on reduction of forward scatter analyses. (Blue = diABZI, orange = UT) **B** STING agonists did not induce significant changes in the frequencies of lymphoid immune subsets. The effect on NKT, T, NK and B cell populations (n = 5 PBMC donors) was analysed as the relative fold difference in the frequency of the respective population between treated samples and UT (UT = 1, dashed lines). Individual donors are represented by different symbols. Data were analysed using the Friedman test with the uncorrected Dunn's test for multiple comparisons. *$P < 0.05$, **$P < 0.01$.

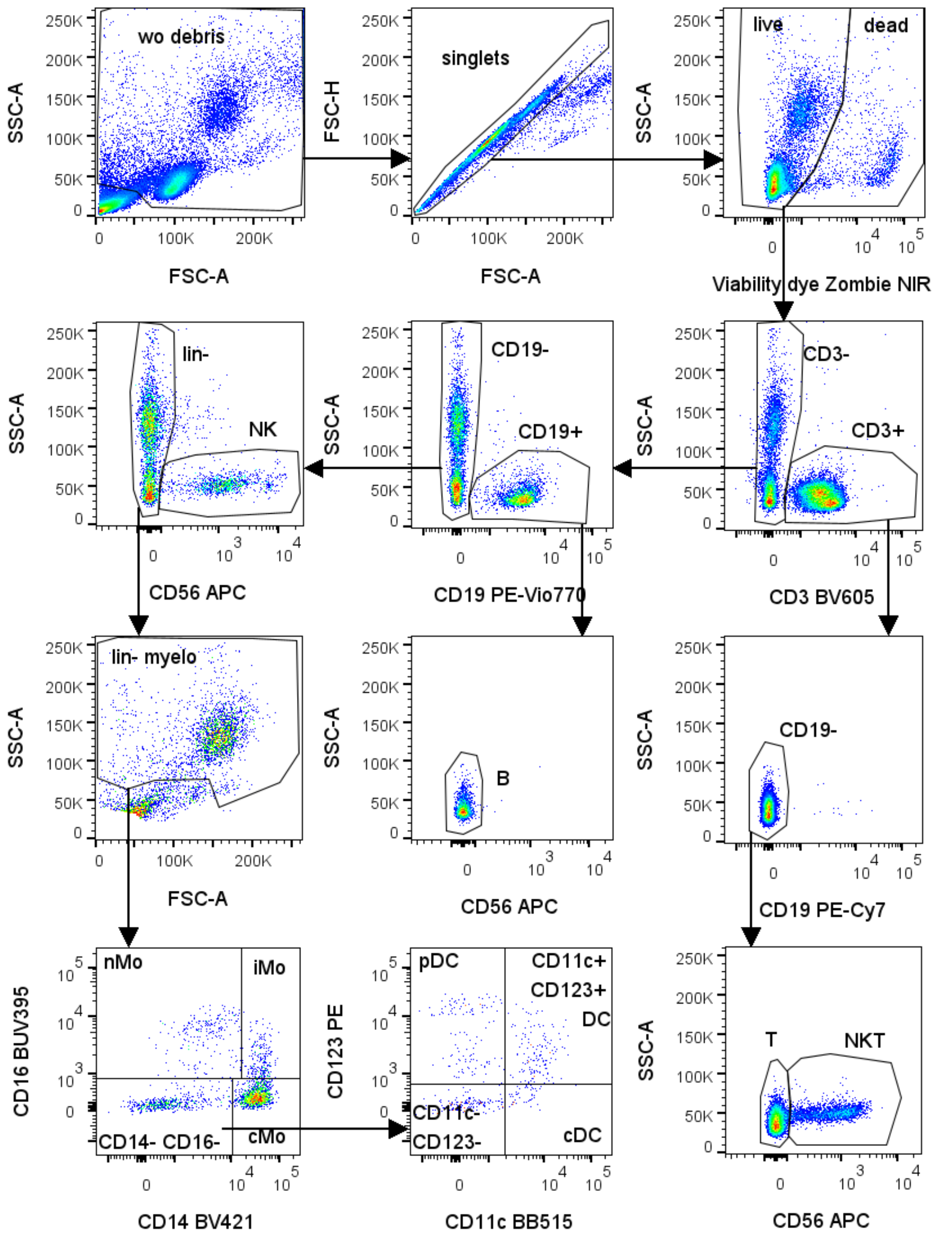

Figure S2: Gating strategy for PBMC immunophenotyping using multiparametric flow cytometry analyses. SSC-A = side scatter – area, FSC-A = forward scatter – area, NIR = near infra-red, NKT = NKT cells, T = T cells, B = B cells, NK = NK cells, lin− = lineage-negative cells CD3−CD19−CD56−, lin− myelo = lin− myeloid subset, nMo = CD14−CD16+ non-classical monocytes, iMo = CD14+ CD16+ intermediate monocytes, cMo = CD14+CD16− classical monocytes, pDC = CD11c−CD123+ plasmacytoid dendritic cell-like cells, CD11c+CD123+ DC = CD11c+CD123+ dendritic cell-like cells, cDC = CD11c+CD123− conventional dendritic cell-like cells.

A

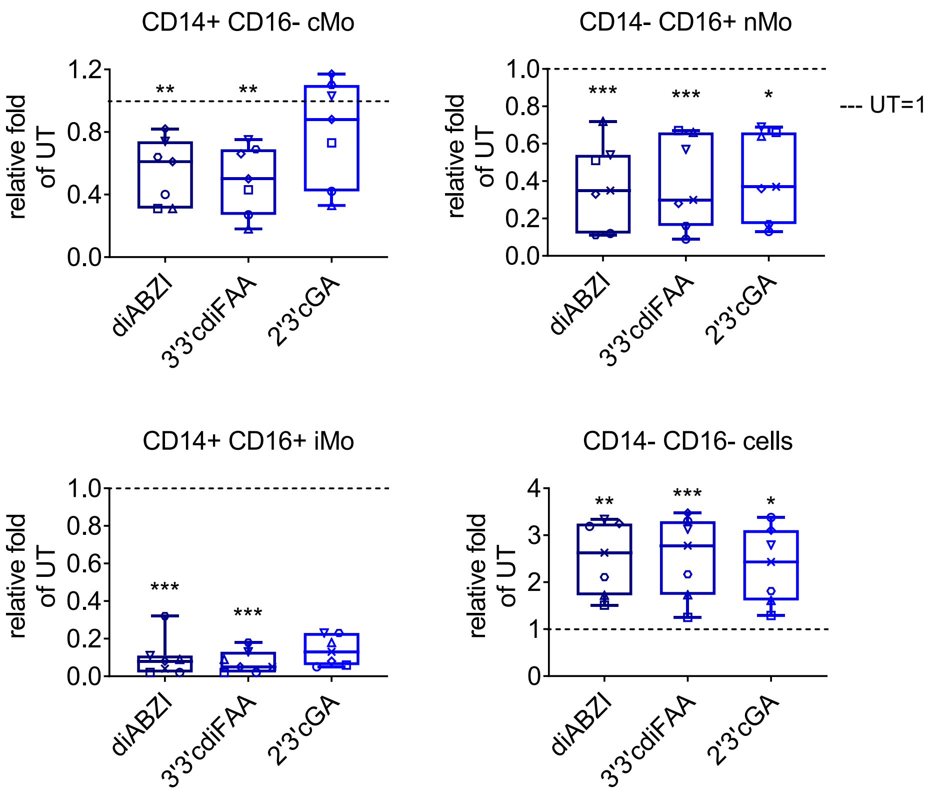

CD14+ CD16- cMo
CD14- CD16+ nMo
CD14+ CD16+ iMo
CD14- CD16- cells
relative fold of UT
--- UT=1
diABZI
3'3'cdiFAA
2'3'cGA
**
***
*

B

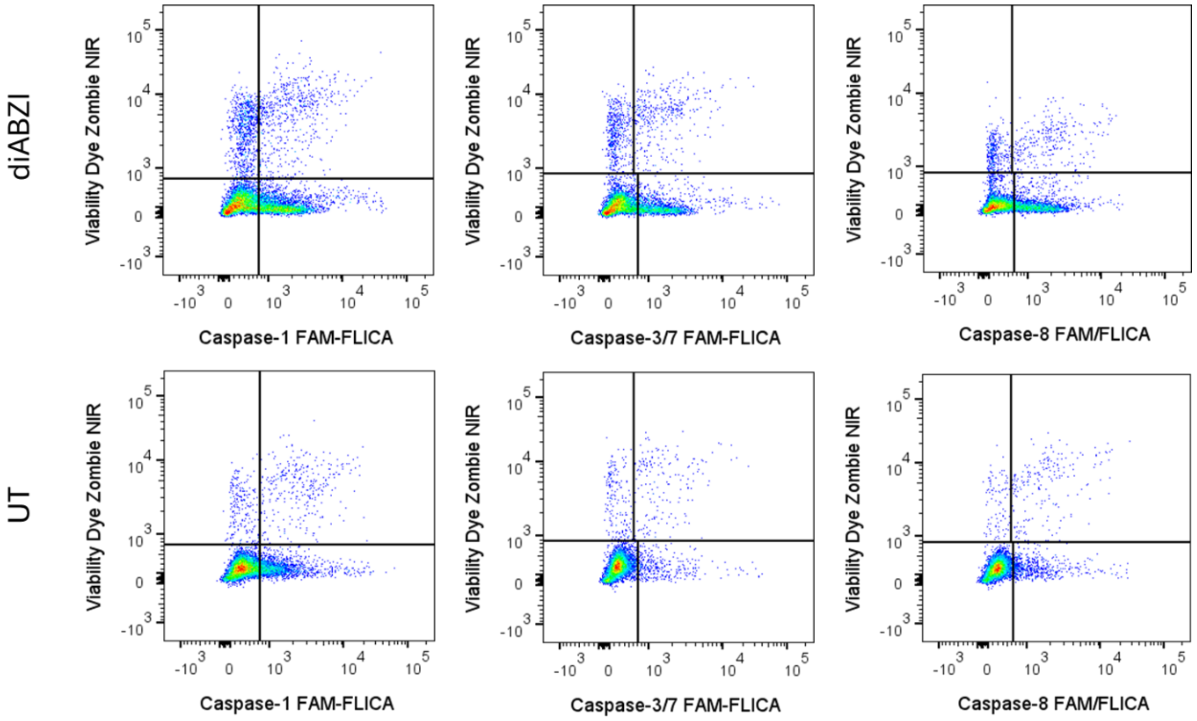

diABZI
UT
Viability Dye Zombie NIR
Caspase-1 FAM-FLICA
Caspase-3/7 FAM-FLICA
Caspase-8 FAM/FLICA

Figure S3: STING agonists induce the activation of apoptotic and pyroptotic caspases in the myeloid subset of PBMCs. **A** Human PBMCs (*n* = 7 donors) were treated for 4 h with the STING agonists: diaminobenzimidazole-based agonist (diABZI), 3′,3′c-di(2′F,2′d-AMP) (3′3′cdiFAA) and 2′,3′-cGAMP (2′3′cGA). Untreated cells (UT) were used as a negative control. The frequency of immune populations was analysed using multiplex immunophenotyping flow cytometry-based analyses. The effect on immune subsets was analysed as the relative fold difference in the frequency of the respective population between treated samples and UT (UT = 1, dashed lines). Individual donors are represented by different symbols. STING agonists induced the depletion of all monocyte populations already upon 4 h of treatment. Data were analysed using the Friedman test with the uncorrected Dunn's test for multiple comparisons. $^{*}P < 0.05$, $^{**}P < 0.01$, $^{***}P < 0.001$. c/i/nMo = classical/intermediate/non-classical monocytes. **B** Human PBMCs (*n* = 7 donors) were treated for 4 h with the STING agonist diaminobenzimidazole-based agonist (diABZI). Untreated cells (UT) were used as a negative control. The activation of caspases was analysed using flow cytometry-based detection of active caspases with FAM-FLICA staining, live/dead staining (Zombie NIR) and multiparametric immunophenotyping. Representative example of the effect of diABZI on the induction of caspase-1, -3/7 and -8 and on live/dead marker positivity.

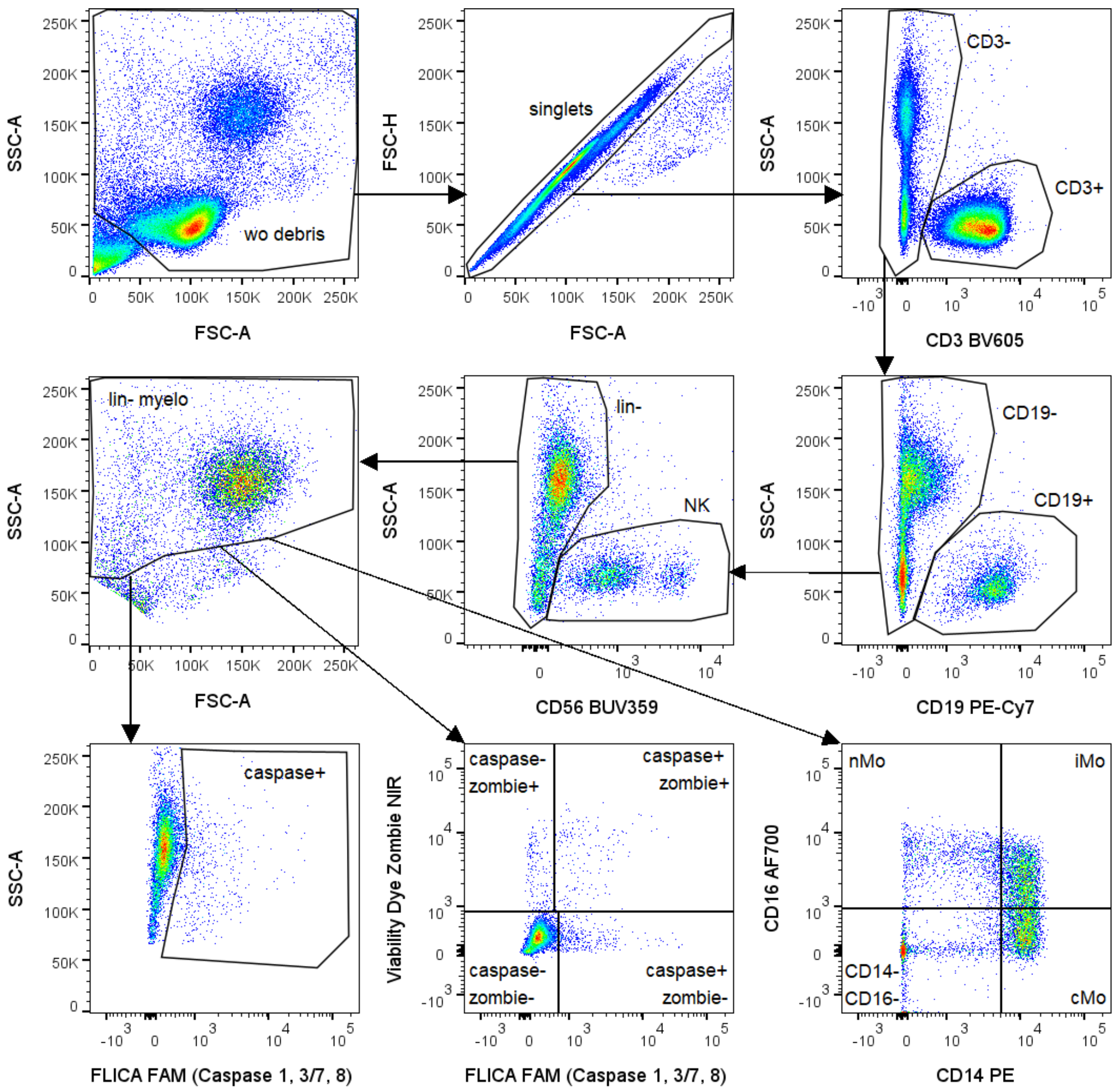

Figure S4: Gating strategy for analyses of caspase activation. SSC-A = side scatter – area, FSC-A = forward scatter – area, NIR = near infra-red, NK = NK cells, lin– = lineage-negative cells CD3–CD19–CD56–, lin– myelo = lin– myeloid subset, nMo = CD14–CD16+ non-classical monocytes, CD14+CD16+ iMo = intermediate monocytes, cMo = CD14+CD16– classical monocytes.

A

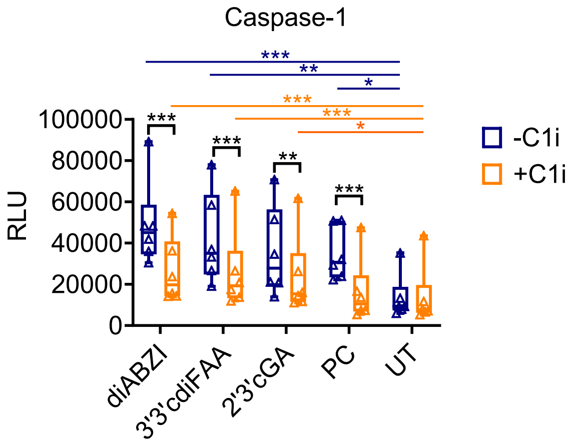

Caspase-1
RLU
100000
80000
60000
40000
20000
0
***
**
*
***
***
*
***
***
**
***
diABZI
3'3'cdiFAA
2'3'cGA
PC
UT
-C1i
+C1i

B

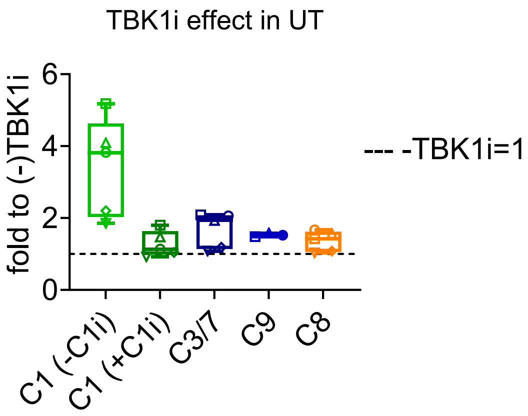

TBK1i effect in UT
fold to (-)TBK1i
6
4
2
0
C1 (-C1i)
C1 (+C1i)
C3/7
C9
C8
--- -TBK1i=1

C

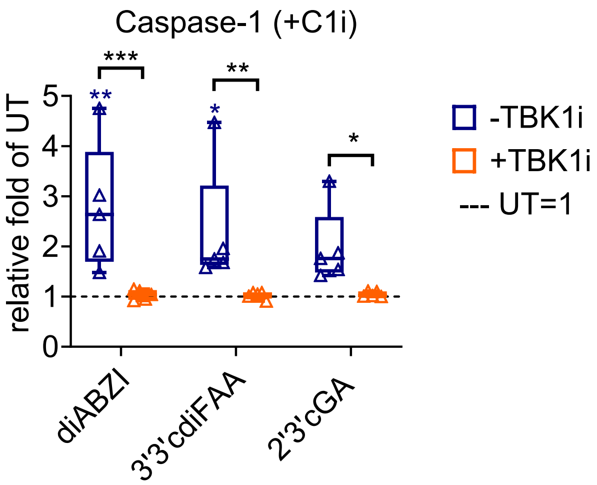

Caspase-1 (+C1i)
relative fold of UT
5
4
3
2
1
0
***
**
*
**
*
diABZI
3'3'cdiFAA
2'3'cGA
-TBK1i
+TBK1i
--- UT=1

D

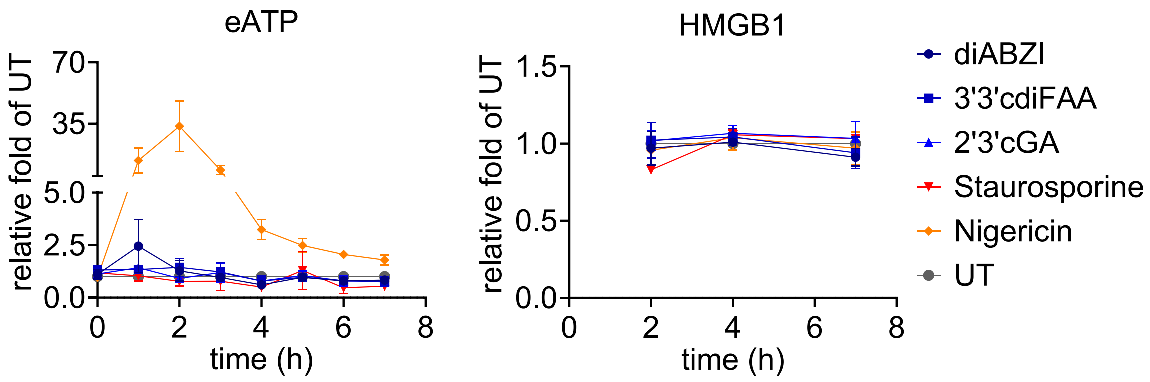

eATP
relative fold of UT
70
35
5.0
2.5
0.0
0
2
4
6
8
time (h)
HMGB1
relative fold of UT
1.5
1.0
0.5
0.0
0
2
4
6
8
time (h)
diABZI
3'3'cdiFAA
2'3'cGA
Staurosporine
Nigericin
UT

Supplementary figure S5: STING agonists induce the activation of apoptotic and pyroptotic caspases in enriched monocytes. **A** Monocytes were enriched from PBMCs ($n = 6$ donors) and treated for 4 h with the STING agonists: diaminobenzimidazole-based agonist (diABZI), 3′,3′c-di(2′F,2′d-AMP) (3′3′cdiFAA) and 2′,3′-cGAMP (2′3′cGA), and the positive control (PC) nigericin. Untreated cells (UT) were used as a negative control. The caspase-1 reporter-based assay is not specific only to caspase-1, but also to caspase-5, -3 and -6. Therefore, a complimentary assay with a caspase inhibitor (C1i, YVAD-CHO) was performed to distinguish between caspase-1 and cross-reacting caspase activity. The residual activity upon caspase-1 inhibition (+C1i) confirms the induction of cross-reacting caspases. RLU = relative luminescence units. The data were analysed using two-way ANOVA to compare −C1i and +C1i and by the Friedman test with the uncorrected Dunn's test for multiple comparisons to compare differences against the respective UT. $*P < 0.05$, $**P < 0.01$, $***P < 0.001$. **B** To demonstrate that the caspase activation was dependent on the activation of the cGAS–STING pathway, enriched monocytes ($n = 3$ - 5 PBMC donors) were pretreated for 30 min with TBK1 inhibitor (TBK1i) MRT68601 hydrochloride followed by 4 h of incubation with or without STING agonists. The effect of the TBK1i on caspase activation was studied in otherwise untreated (UT) monocytes ($n = 5$ PBMC donors). Individual donors are represented by different symbols. The dashed line represents the baseline frequency of untreated −TBK1i monocytes (−TBK1i = 1). The data were analysed using Wilcoxon matched-pairs signed rank test with no statistically significant results. −/+C1i = +/−caspase-1 inhibitor (YVAD-CHO) described in part A. **C** A complimentary caspase-1 assay for residual activity of cross-reacting caspases described in part A was performed also for the TBK1i assay described in part B. Upon 30 min of TBKi pretreatment, enriched monocytes were treated for 4 h with the STING agonists: diaminobenzimidazole-based agonist (diABZI), 3′,3′c-di(2′F,2′d-AMP) (3′3′cdiFAA) and 2′,3′-cGAMP (2′3′cGA). Untreated cells (UT) were used as a negative control. TBK1i inhibits cross-reacting caspases in the caspase-1 reporter assay. The data were analysed using two-way ANOVA to compare −TBK1i and +TBK1i and by the Friedman test with the uncorrected Dunn's test for multiple comparisons to compare differences against the respective

untreated cells (UT). $*P < 0.05$, $**P < 0.01$, $***P < 0.001$. **D** Enriched monocytes ($n = 3$ donors) were continuously incubated with the STING agonists: diaminobenzimidazole-based agonist (diABZI), 3′,3′c-di(2′F,2′d-AMP) (3′3′cdiFAA) and 2′,3′-cGAMP (2′3′cGA) and the positive control (PC) nigericin. Untreated cells (UT) were used as a negative control. Analyses of DAMPs (extracellular ATP (eATP) and secreted HMGB1 protein) were monitored over time using reporter-based assays. The data represent means of biological replicates ± the standard error of the mean.

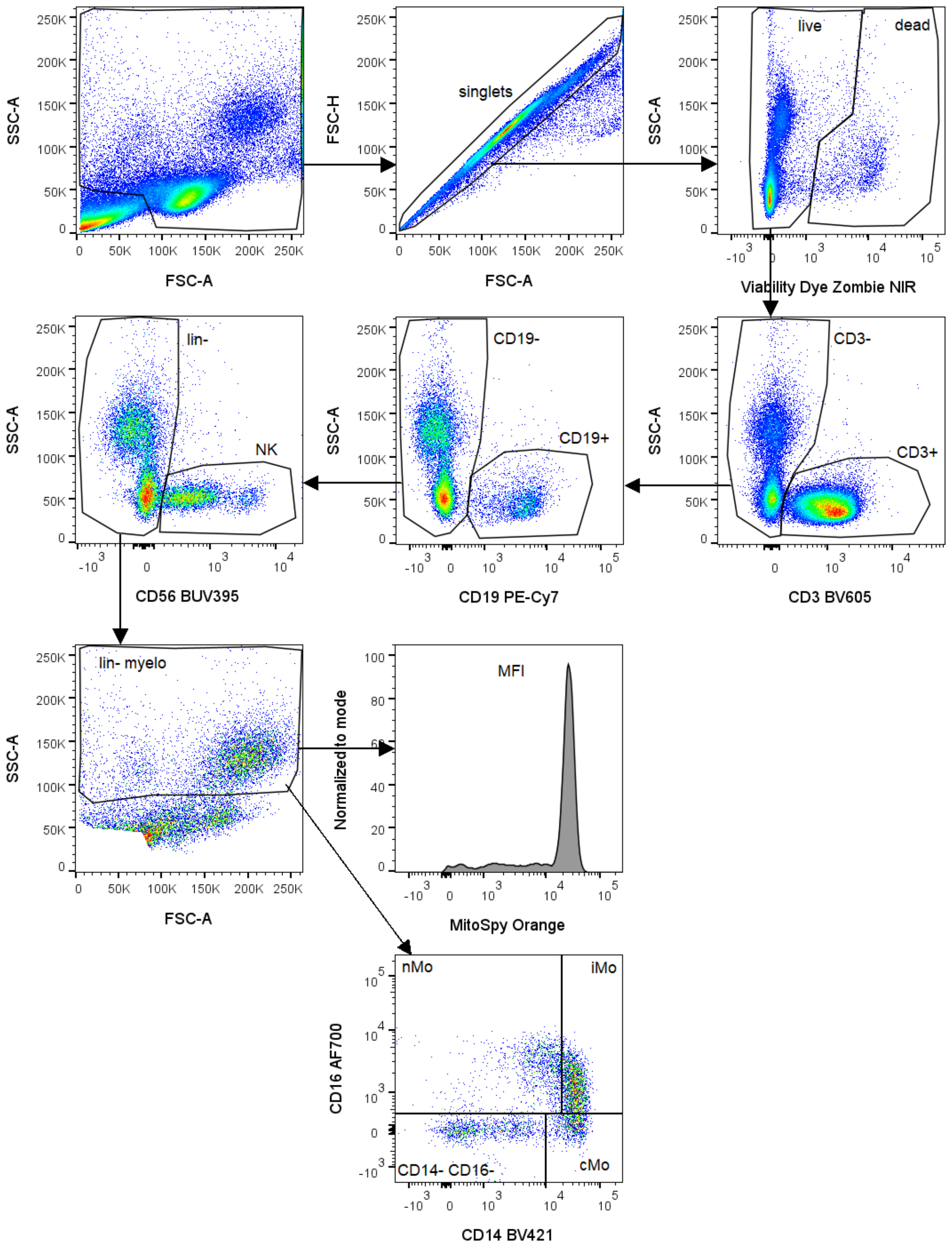

Figure S6: Gating strategy for analyses of mitochondrial fitness. SSC-A = side scatter – area, FSC-A = forward scatter – area, NIR = near infra-red, NK = NK cells, lin− = lineage-negative cells CD3−CD19−CD56−, lin− myelo = lin− myeloid subset, MFI = median fluorescence intensity, nMo = CD14−CD16+ non-classical monocytes, iMo = CD14+CD16+ intermediate monocytes, cMo = CD14+CD16− classical monocytes.

A

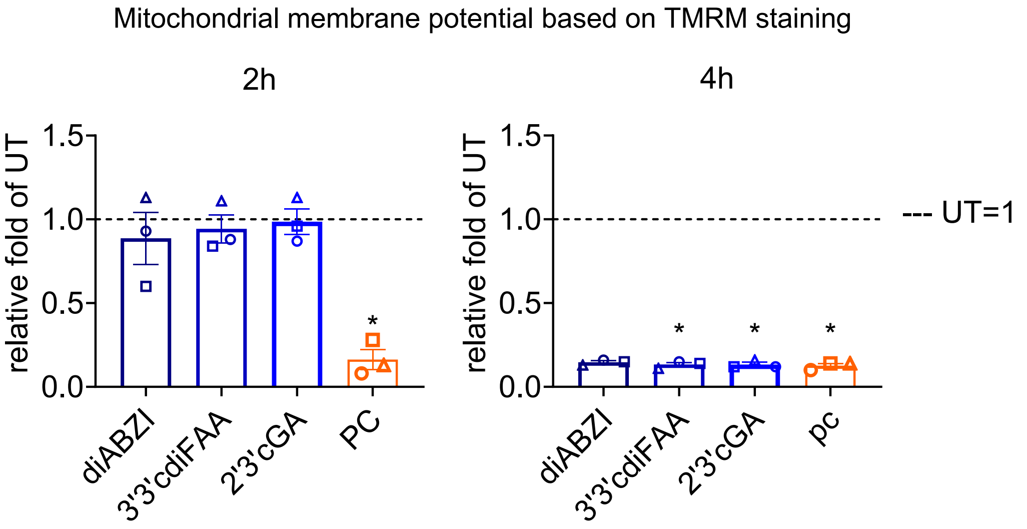
Mitochondrial membrane potential based on TMRM staining
2h
4h
relative fold of UT
1.5
1.0
0.5
0.0
--- UT=1
diABZI
3'3'cdiFAA
2'3'cGA
PC
pc
*

B

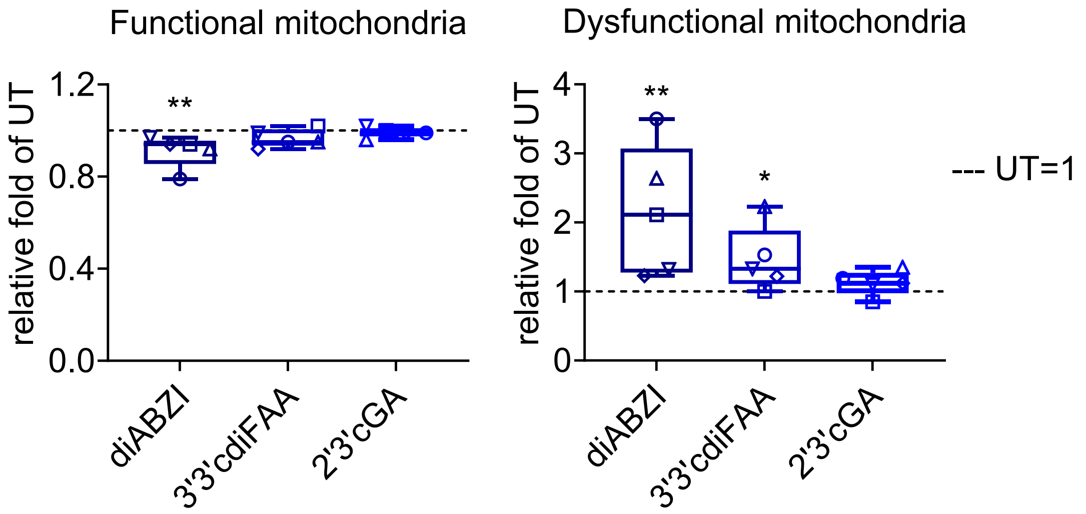
Functional mitochondria
Dysfunctional mitochondria
relative fold of UT
1.2
0.8
0.4
0.0
4
3
2
1
0
**
*
--- UT=1
diABZI
3'3'cdiFAA
2'3'cGA

C

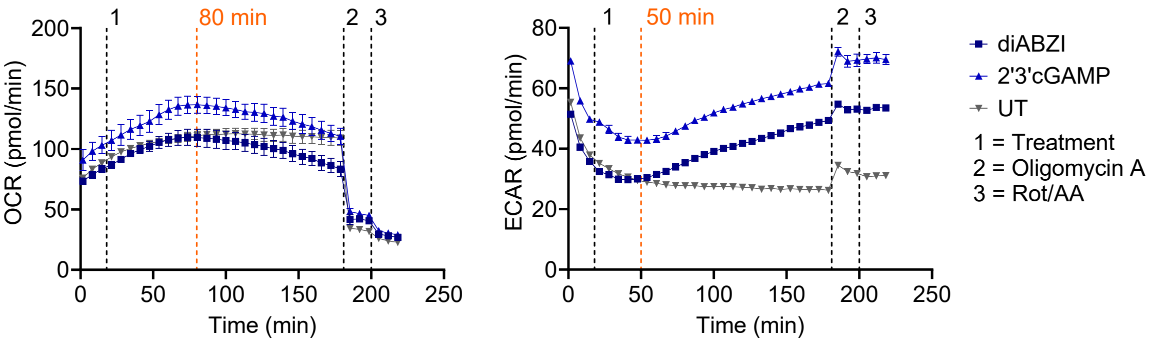
OCR (pmol/min)
ECAR (pmol/min)
Time (min)
80 min
50 min
1
2
3
diABZI
2'3'cGAMP
UT
1 = Treatment
2 = Oligomycin A
3 = Rot/AA

Supplementary figure S7: STING agonists induce mitochondrial dysfunction and alter the metabolism of monocytes. **A** The effect of STING agonists on mitochondrial membrane potential was determined as the change in median fluorescence intensity (MFI) of TMRM staining in enriched monocytes (n = 3 PBMC donors) treated for 2 and 4 h with diaminobenzimidazole-based agonist (diABZI), 3′,3′c-di(2′F,2′d-AMP) (3′3′cdiFAA) and 2′,3′-cGAMP (2′3′cGA). Staurosporine was used as a positive control (PC). Untreated cells (UT) were used as a control. The effect of compounds on mitochondrial membrane potential of enriched monocytes is presented as the relative fold difference in the TMRM dye MFI between treated samples and UT (UT=1, dashed lines). Individual donors are represented by different symbols. The data were analysed using the Friedman test with the uncorrected Dunn's test for multiple comparisons. $*P < 0.05$. **B** The effect of STING agonists on the mitochondrial function of enriched monocytes was determined using MitoTracker Green FM and Deep Red FM staining. Enriched monocytes ($n = 5$ PBMC donors) were treated for 2 h with the diaminobenzimidazole-based agonist (diABZI), 3′,3′c-di(2′F,2′d-AMP) (3′3′cdiFAA) and 2′,3′-cGAMP (2′3′cGA). Untreated cells (UT) were used as a control. The effect of STING agonists on mitochondrial function of enriched monocytes is presented as the relative fold difference in the frequency of monocytes with functional/dysfunctional mitochondria between treated samples and UT (UT=1, dashed lines). Individual donors are represented by different symbols. The data were analysed using the Friedman test with the uncorrected Dunn's test for multiple comparisons. $*P < 0.05$, $**P < 0.01$. **C** Effect of selected STING agonists: 3′,3′c-di(2’F,2’d-AMP) (3’3’cdiFAA) and 2′,3’-cGAMP (2’3’cGA) on the oxygen consumption rate (OCR) and extracellular acidification rate (ECAR), measured using the Seahorse analyser (representative result for one PBMC donor). Orange vertical lines mark the onset of STING agonist effects. Rot/AA = rotenone + antimycin A mix.

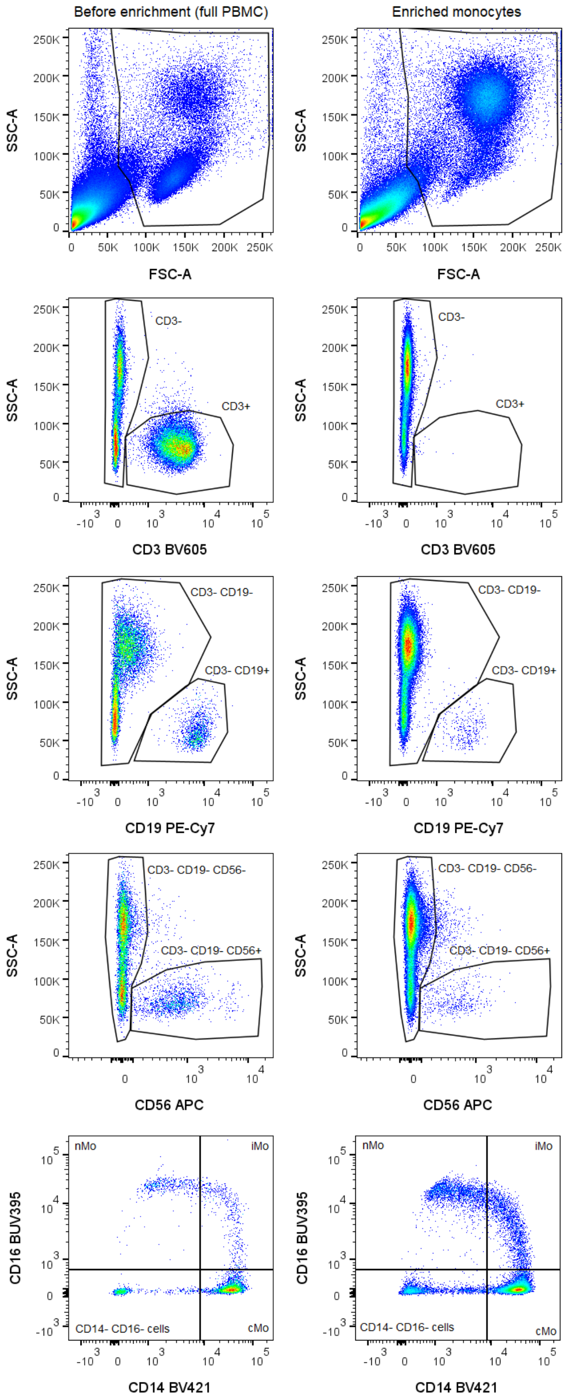

Supplementary Fig. S8: Representative example of enriched monocyte purity determination using the gating strategy presented in Fig. S2. The isolation effectively enriched the monocyte populations with minor contamination of B and NK cells and residual DC-like cells, which cannot be separated from monocytes using this method. n/i/cMo = non-classical/intermediate/classical monocytes.

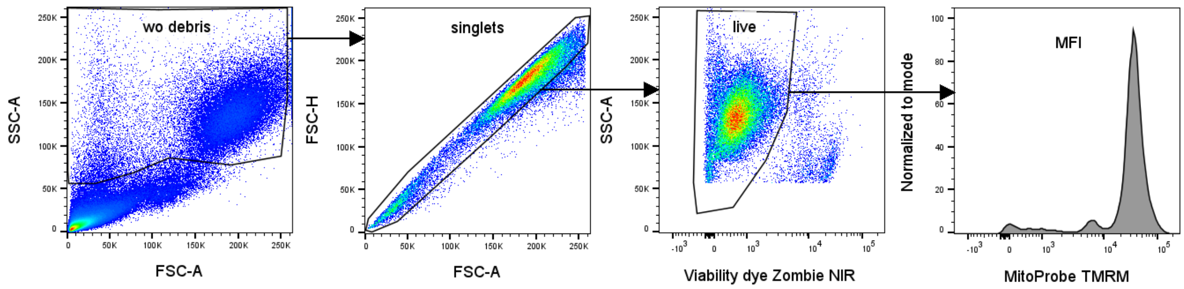

Figure S9: Gating strategy for analyses of mitochondrial membrane potential using TMRM dye. SSC-A = side scatter – area, FSC-A = forward scatter – area, NIR = near infrared, MFI = median fluorescence intensity

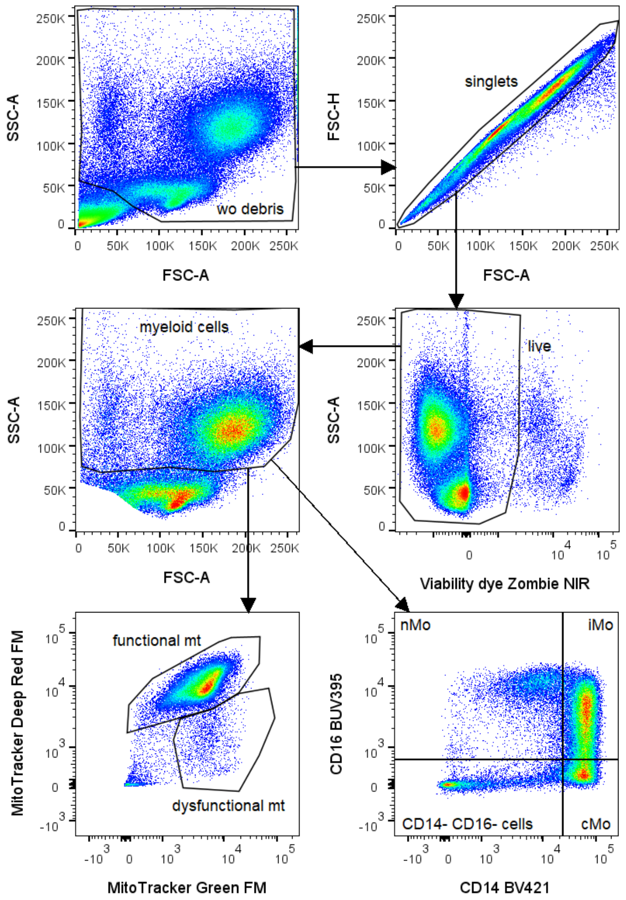

Figure S10: Gating strategy for analyses of mitochondrial dysfunction. SSC-A = side scatter – area, FSC-A = forward scatter – area, NIR = near infra-red, mt = mitochondria, nMo = CD16+CD14– non-classical monocytes, iMo = CD14+CD16+ intermediate monocytes, cMo = CD14+CD16– classical monocytes.

Table S1: Flow cytometry panel for general PBMC immunophenotyping.

Antibody	Catalogue number	Clone	Isotype	Manufacturer
mouse anti-human CD16 BUV395	563784	3G8	IgG1 κ	BD Biosciences, Franklin Lakes, USA
mouse anti-human CD3 BV605	563219	SK7	IgG1 κ	
mouse anti-human CD14 BV421	565283	M5E2	IgG2a κ	
mouse anti-human CD11c BB515	564490	B-ly6	IgG1 κ	
recombinant human anti-human CD19 PE-Vio770	130-113-647	REA675	IgG1	Miltenyi Biotec (Bergisch Gladbach, Germany)
mouse anti-human CD123 PE	566919	6H6	IgG1 κ	BD Biosciences
mouse anti-human CD11b Alexa Fluor 700*	557918	ICRF44	IgG1 κ	
mouse anti-human CD56 APC	341027	NCAM16.2	IgG2b κ	
Zombie NIR viability dye	423106	n/a	n/a	BioLegend (San Diego, California, USA)

* Used for unbiased multiparametric analyses.

Table S2: Flow cytometry panel for analyses of caspase activation.

**Antibody**	Catalogue number	**Clone**	**Isotype**	**Manufacturer**
mouse anti-human CD56 BUV395	563554	NCAM16.2	IgG2b κ	BD Biosciences
mouse anti-human CD3 BV605	563219	SK7	IgG1 κ	
FAM-FLICA caspase-3/7 or caspase-1 or caspase-8	ICT094 or ICT9146 or APT408	n/a	n/a	BioRad Hercules, California, USA (caspase-3/7, caspase-1)/Merck Rahway, New Jerzy, USA (caspase-8)
mouse anti-human CD19 PE-Cy7	557835	SJ25C	IgG1 κ	BD Biosciences
mouse anti-human CD14 PE	50-0149-T025	61D3	IgG1 κ	Tonbo Biosciences (San Diego, California, USA)
mouse anti-human CD16 Alexa Fluor 700	557920	3G8	IgG1 κ	BD Biosciences
Zombie NIR viability dye	423106	n/a	n/a	BioLegend

Table S3: Flow cytometry panel for analyses of mitochondrial fitness.

Antibody	Catalogue number	Clone	Isotype	Manufacturer
mouse anti-human CD56 BUV395	563554	NCAM16.2	IgG2b κ	BD Biosciences
mouse anti-human CD3 BV605	563219	SK7	IgG1 κ	
mouse anti-human CD14 BV421	565283	M5E2	IgG2a κ	
mouse anti-human CD19 PE-Cy7	557835	SJ25C	IgG1 κ	
MitoSpy Orange CMTMRos	424803	n/a	n/a	BioLegend
mouse anti-human CD16 Alexa Fluor 700	557920	3G8	IgG1 κ	BD Biosciences
Zombie NIR viability dye	423106	n/a	n/a	BioLegend

Table S4: Flow cytometry panel for analyses of mitochondrial potential.

Antibody	Catalogue number	Clone	Isotype	Manufacturer
MitoProbe TMRM Kit	M20036	n/a	n/a	ThermoFisher Scientific, Waltham, Massachusetts, USA
Zombie NIR viability dye	423106	n/a	n/a	BioLegend

Table S5: Flow cytometry panel for analyses of mitochondrial dysfunction.

Antibody	Catalogue number	Clone	Isotype	Manufacturer
mouse anti-human CD16 BUV395	563784	3G8	IgG1 κ	BD Biosciences
mouse anti-human CD14 BV421	565283	M5E2	IgG2a κ	
MitoTracker Green FM Dye	M7514	n/a	n/a	ThermoFisher Scientific, ~~Waltham, Massachusetts, USA~~
MitoTracker Deep Red FM Dye	M22426	n/a	n/a	
Zombie NIR viability dye	423106	n/a	n/a	BioLegend

Table S6: List of antibodies for immunodetection and the respective solutions used.

Antibody	Catalogue number	Clone	Manufacturer
mouse anti-human β-actin	A5441	AC-15	Merck (Rahway, New Jersey, USA)
rabbit anti-human caspase-1	3866S	D7F10	Cell Signaling Technology (Danvers, Massachusetts, USA)
rabbit anti-human caspase-3	14220S	D3R6Y	
rabbit anti-human caspase-7	12827S	D2Q3L	
rabbit anti-human caspase-8	4790S	D35G2	
rabbit anti-human gasdermin D	97558S	E8G3F	
rabbit anti-human RIPK1	3493S	D94C12	
rabbit anti-human phospho-RIPK1 (Ser166)	65746S	D1L3S	
rabbit anti-human RIPK3	10188S	E1Z1D	
rabbit anti-human MLKL	14993S	D2I6N	
Rabbit anti-human phospho-RIPK3 (Ser227)	ab209384	EPR9627	Abcam (Cambridge, UK)
rabbit anti-human phospho-MLKL (Ser358)	ab187091	EPR9514	
IRDye 800CW goat anti-mouse IgG (polyclonal)	926-32210	n/a	Li-cor (Biosciences, Lincoln, Nebrasca, USA)
IRDye 800CW goat anti-rabbit IgG (polyclonal)	926-32211	n/a	
