# Supplementary material for: STING agonists trigger monocyte death via apoptosis, pyroptosis, caspase-8 activation and mitochondrial dysfunction
Source: Cell Death Discov. 2025 Oct 31;11:494. doi: 10.1038/s41420-025-02786-1 (PMC12579220; doi:10.1038/s41420-025-02786-1)

STING Agonists Trigger Monocyte Death via Apoptosis, Pyroptosis, Caspase-8 Activation and Mitochondrial Dysfunction

Marketa Pimkova Polidarova[1,2], Lydie Plecita-Hlavata[3], Ivan Hirsch[1,2], Klara Grantz Saskova[1*], Andrea Brazdova[1*]

[1] Department of Genetics and Microbiology, Faculty of Science, Charles University, BIOCEV, Vestec, Czech Republic

[2] Institute of Organic Chemistry and Biochemistry of the Czech Academy of Sciences, Prague, Czech Republic

[3] Laboratory of Pancreatic Islet Research, Institute of Physiology of the Czech Academy of Sciences, Prague, Czech Republic

* Corresponding authors: andrea.brazdova@natur.cuni.cz (A.B.); saskova2@natur.cuni.cz (K.G.S.)

Supporting information – Original full-length western blots

β-actin

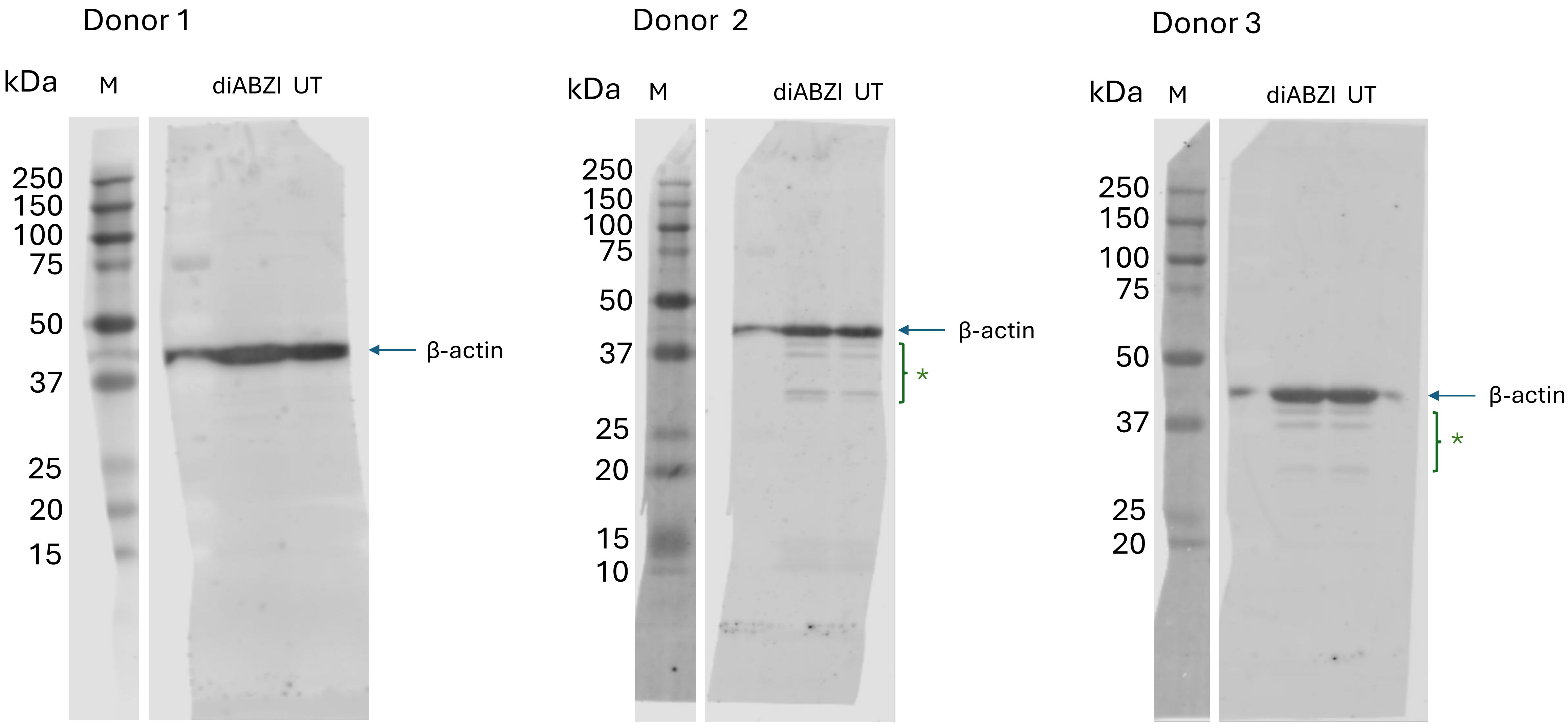

* Unknown bands, likely actin degradation products

β-actin

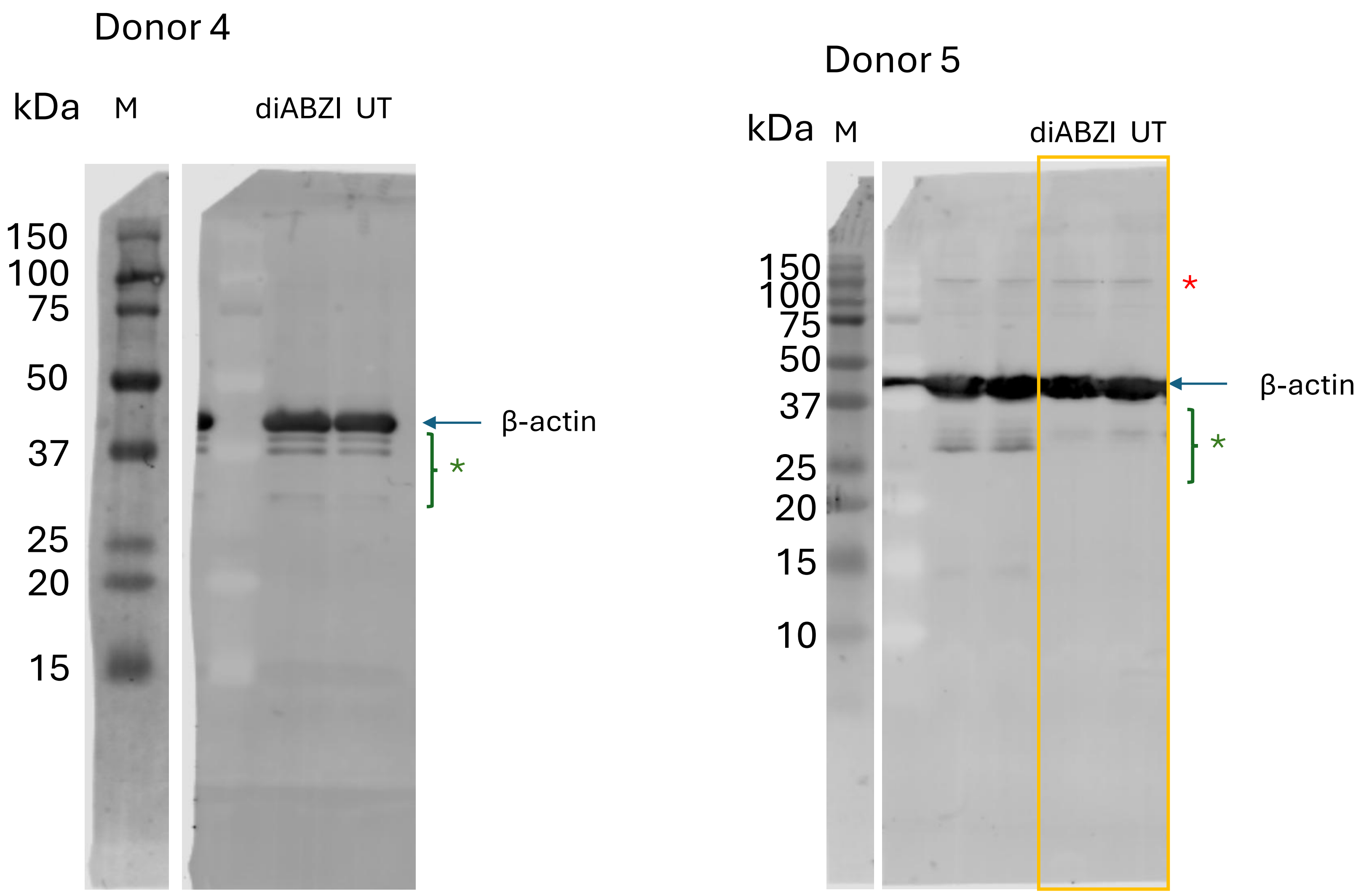

*Unknown bands, likely actin degradation products

* Unknown band

Area of interest

Caspase-1

FL = full length procaspase
p20 = active caspase
* Unknown nonspecific bands

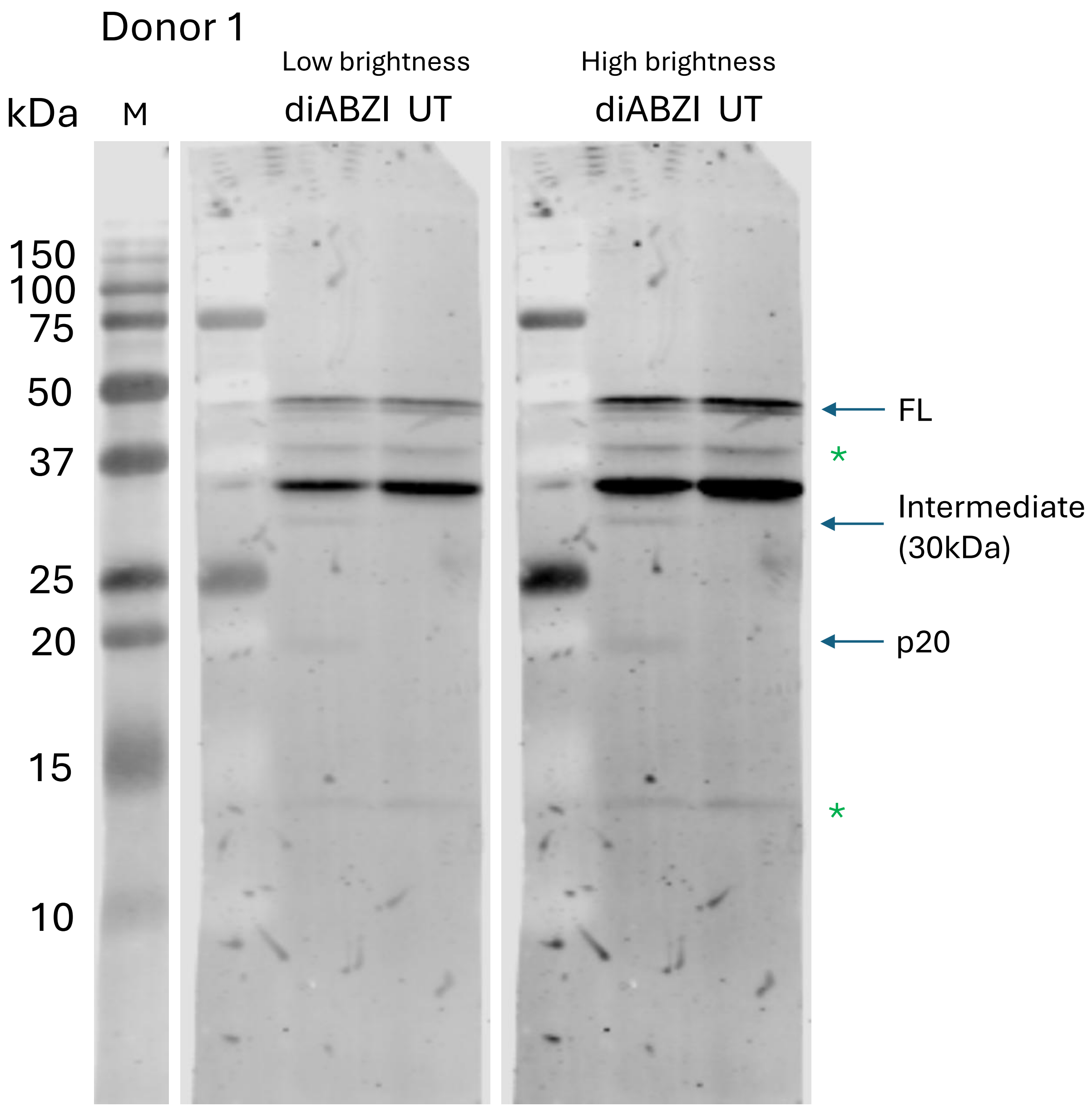

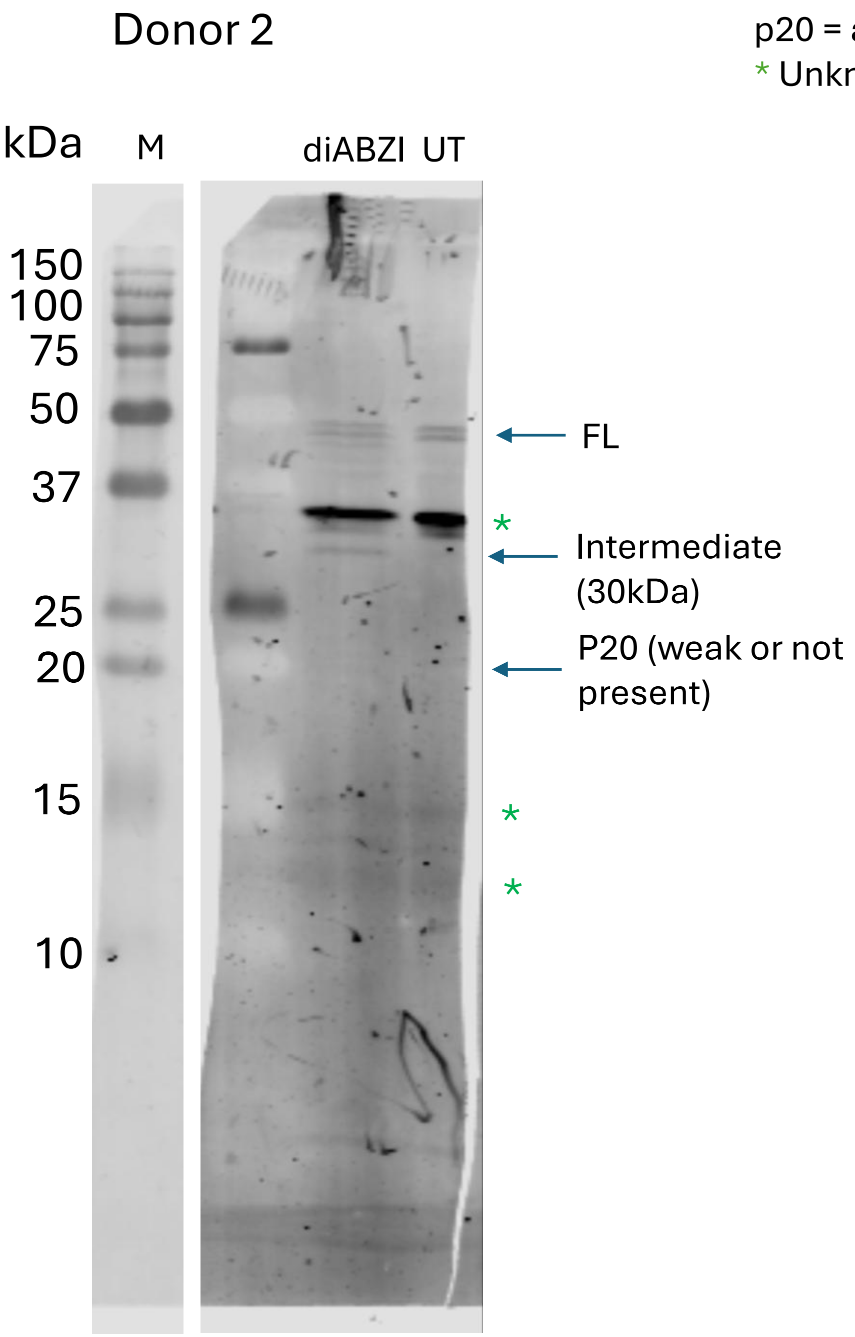

Caspase-1

FL = full length procaspase
p20 = active caspase
* Unknown nonspecific bands

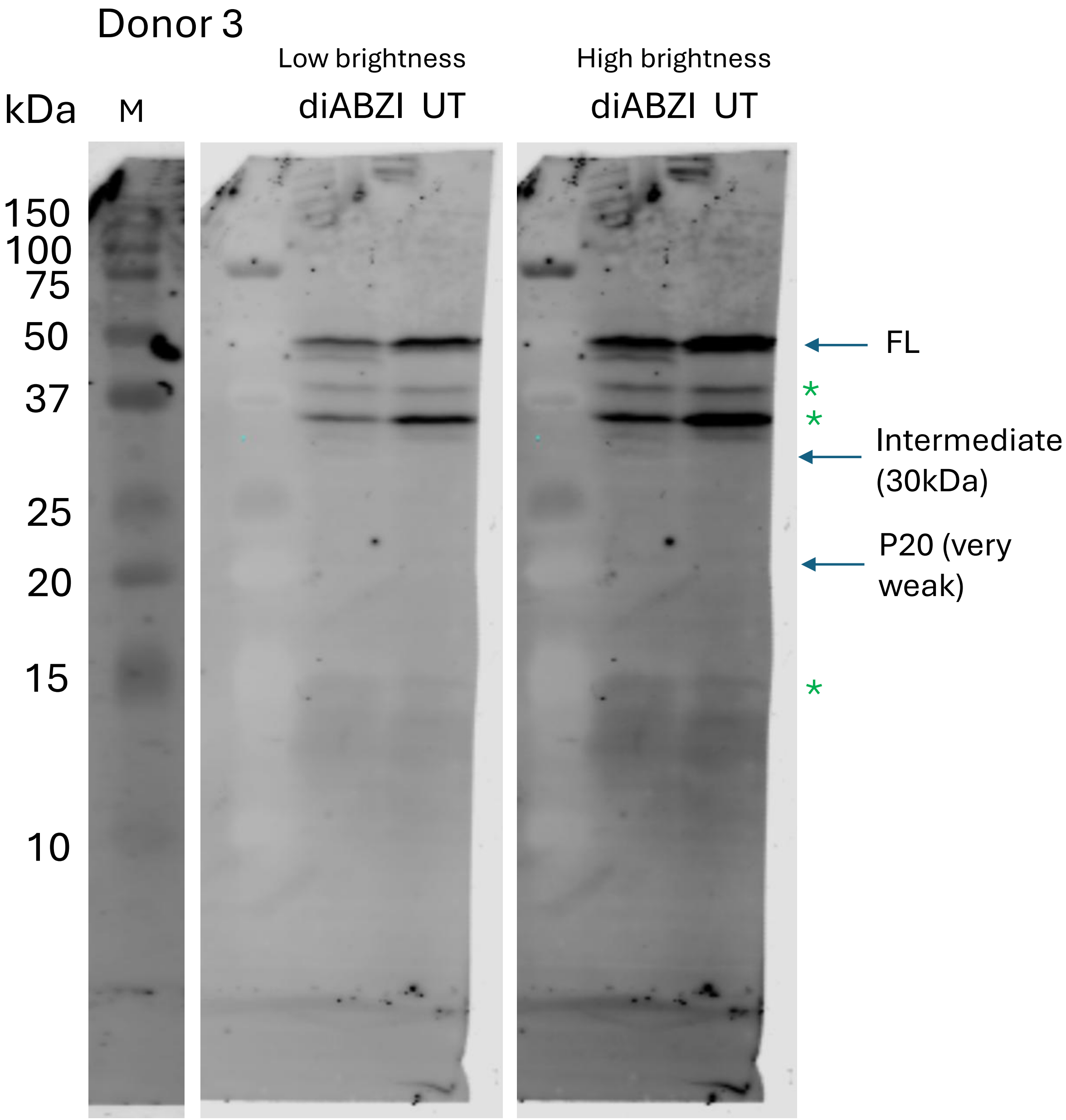

Caspase-1

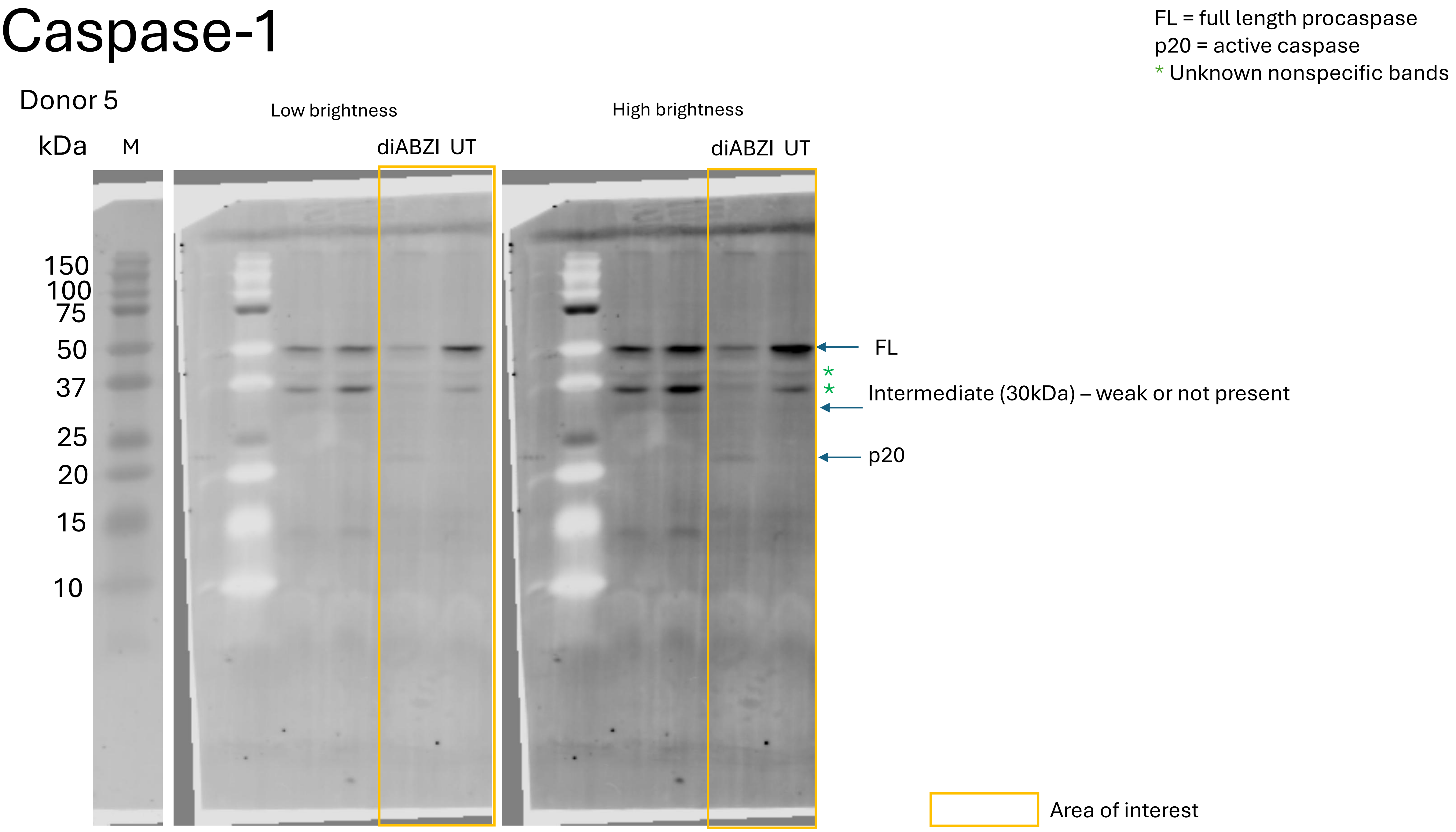
FL = full length procaspase
p20 = active caspase
* Unknown nonspecific bands
Donor 5
Low brightness
High brightness
kDa
M
diABZI
UT
diABZI
UT
150
100
75
50
37
25
20
15
10
FL
*
*
Intermediate (30kDa) – weak or not present
p20
Area of interest

GSDMD

FL = full lenght
p43 = cleaved by caspase 3/7
p30 = cleaved by caspase 1
p21 = cleaved by capsase 1 and 3/7
* Unknown nonspecific bands

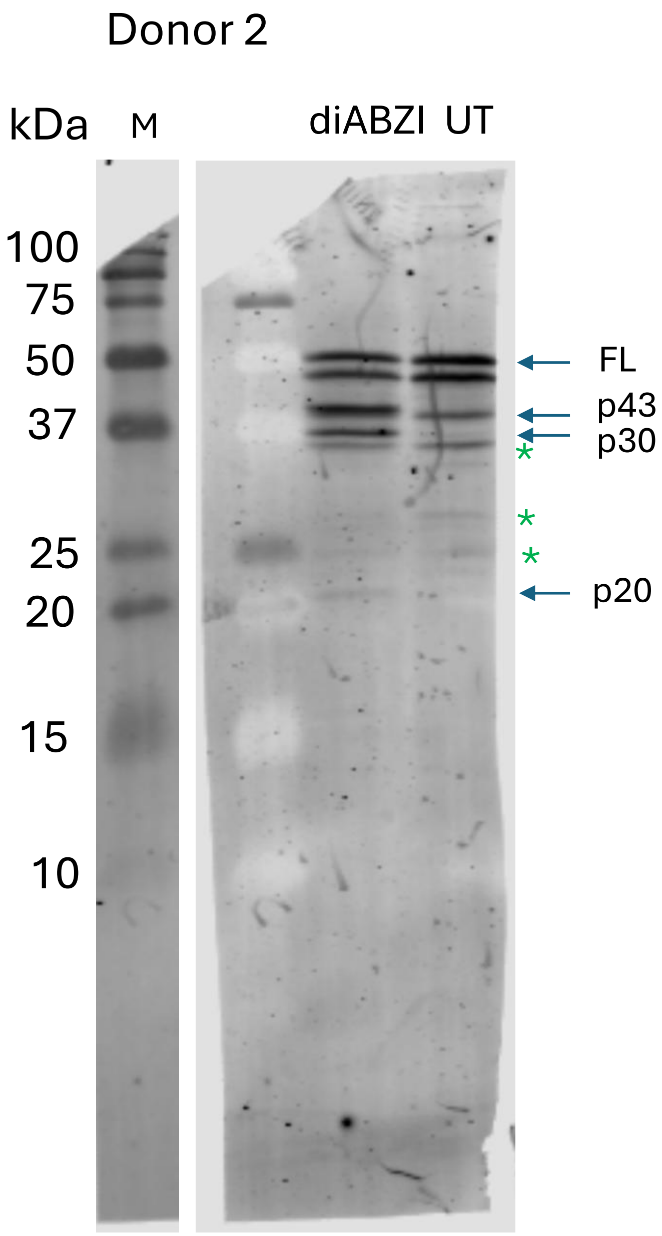

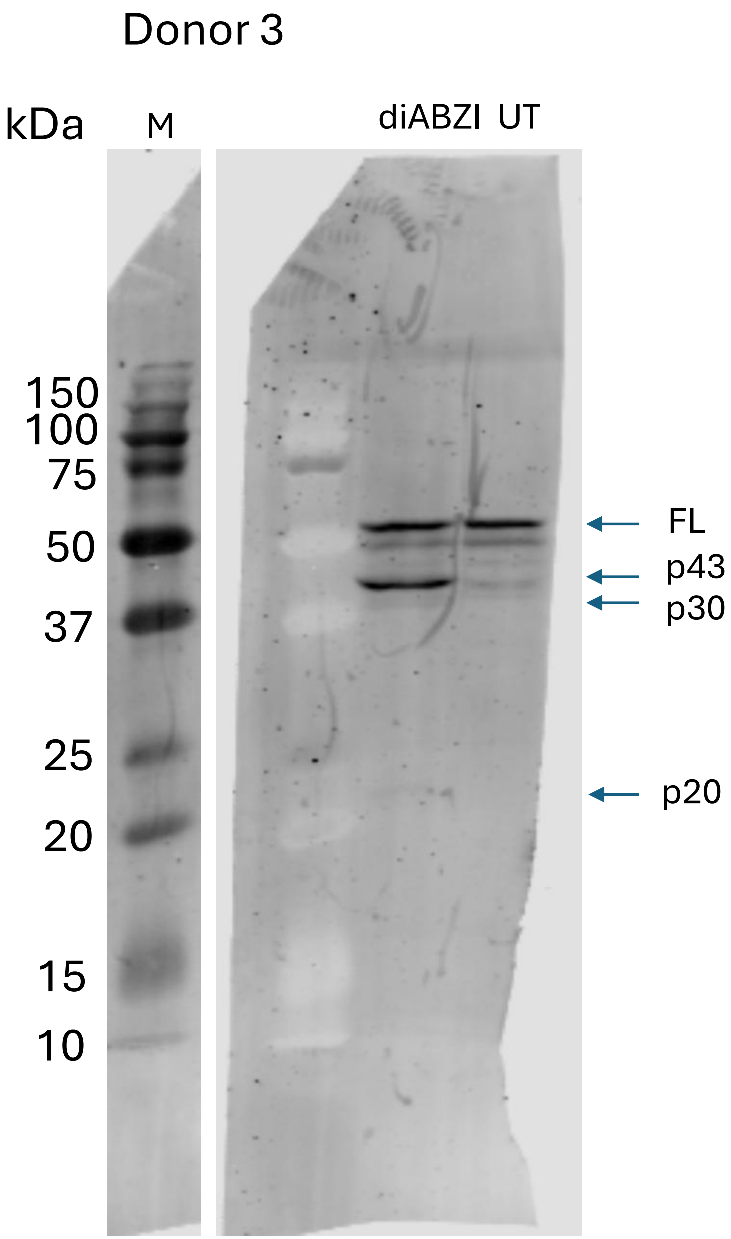

GSDMD

FL = full length
p43 = cleaved by caspase 3/7
p30 = cleaved by caspase 1
p21 = cleaved by capsase 1 and 3/7
* Unknown nonspecific bands

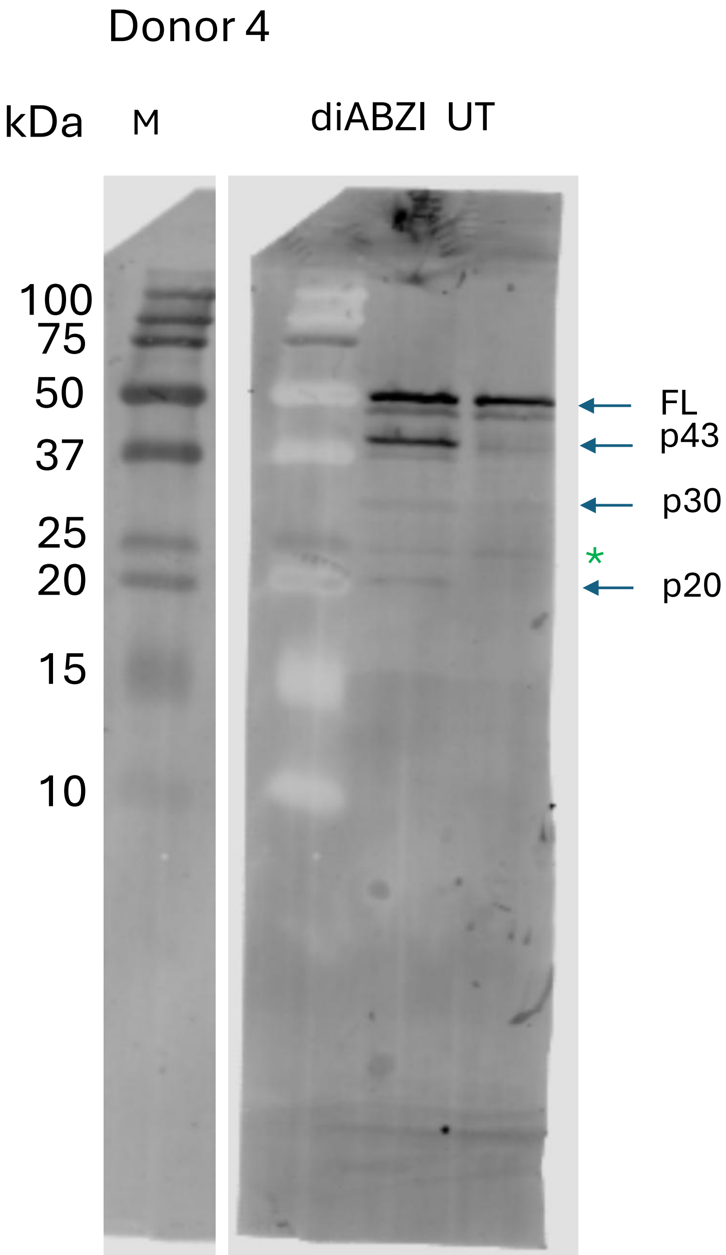

Caspase-3

FL = full length
p19, p17 = active caspase
* Unknown nonspecific bands

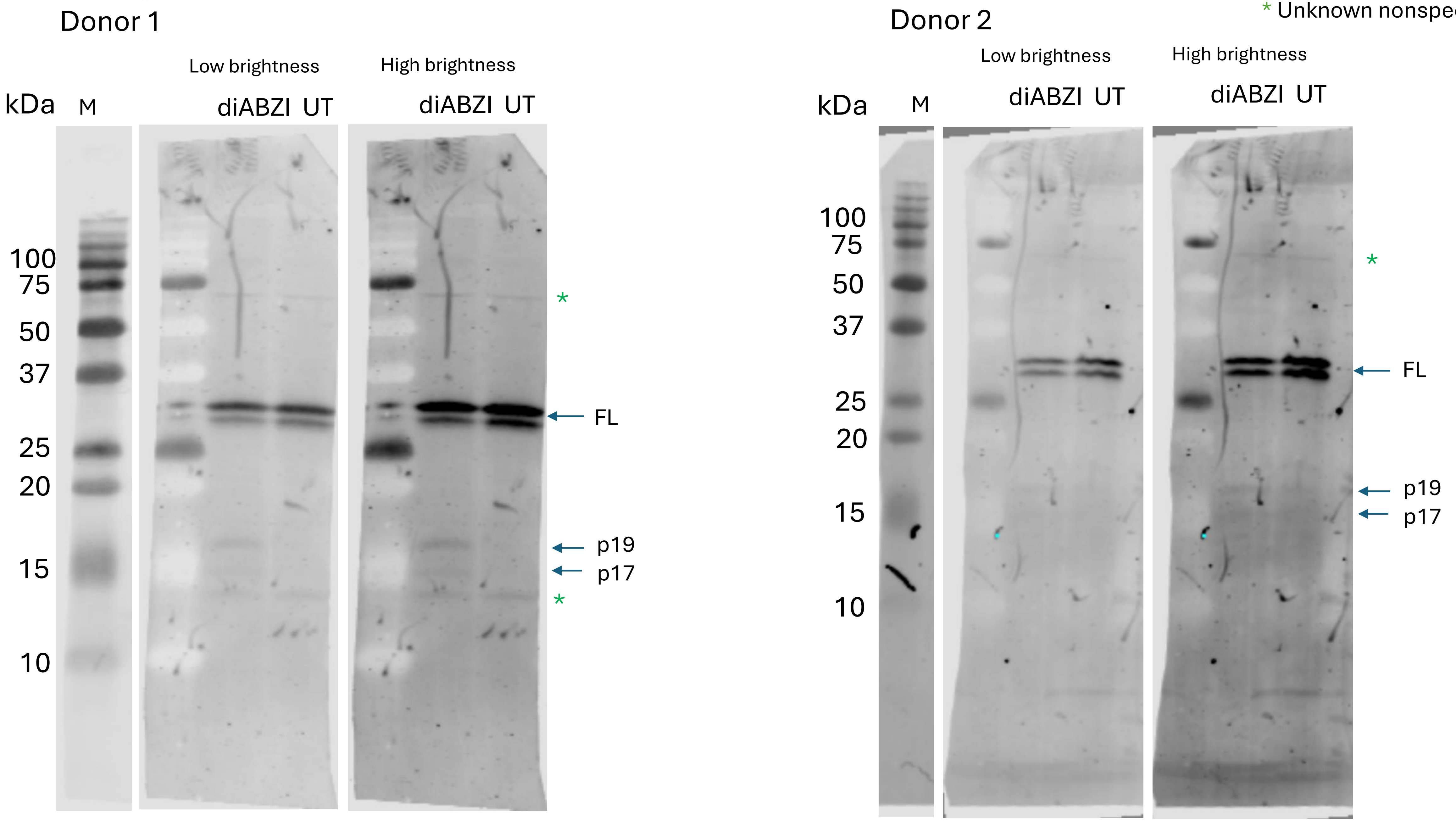

Caspase-3

FL = full length
p19, p17 = active caspase
* Unknown nonspecific bands

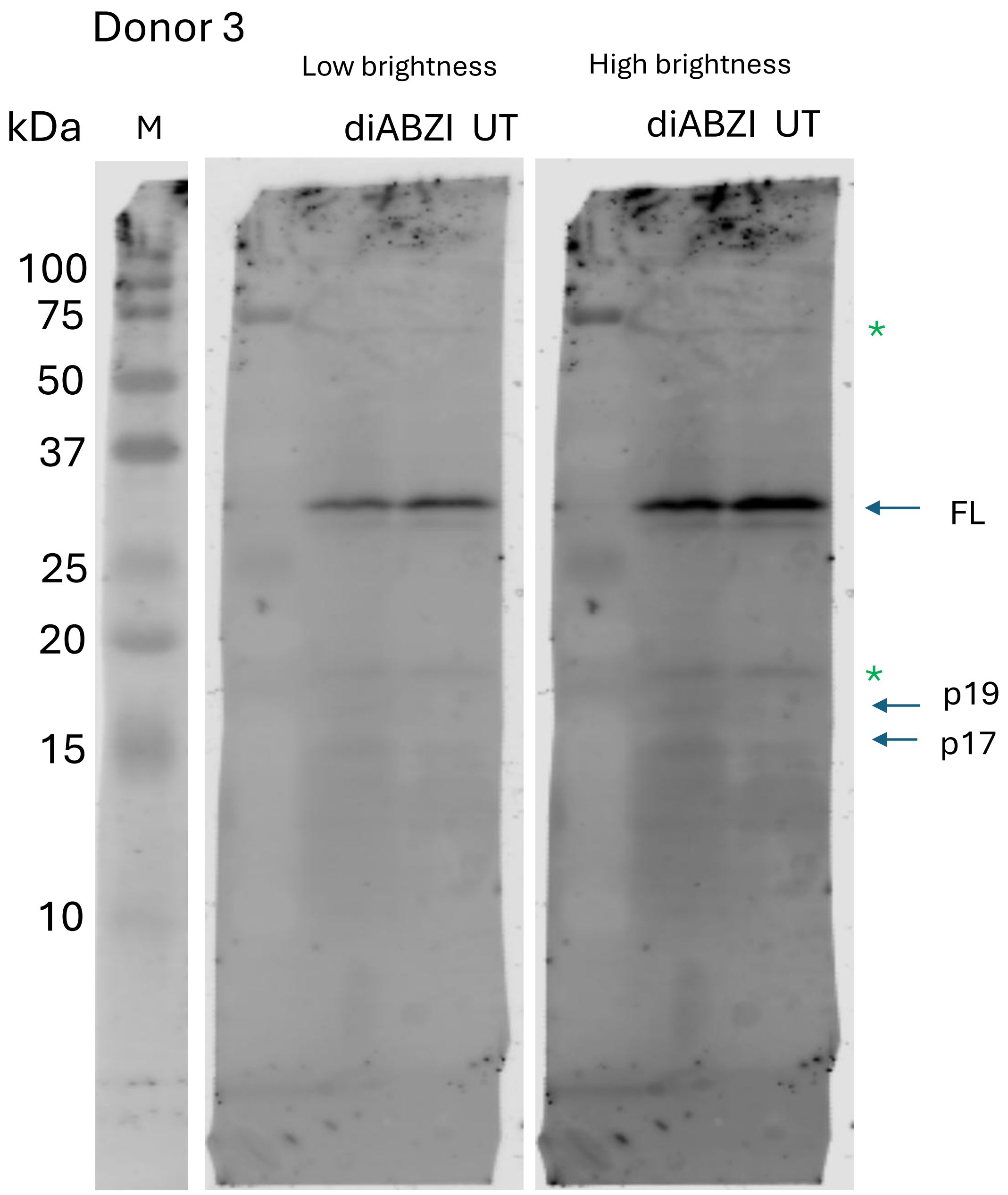

Caspase-7

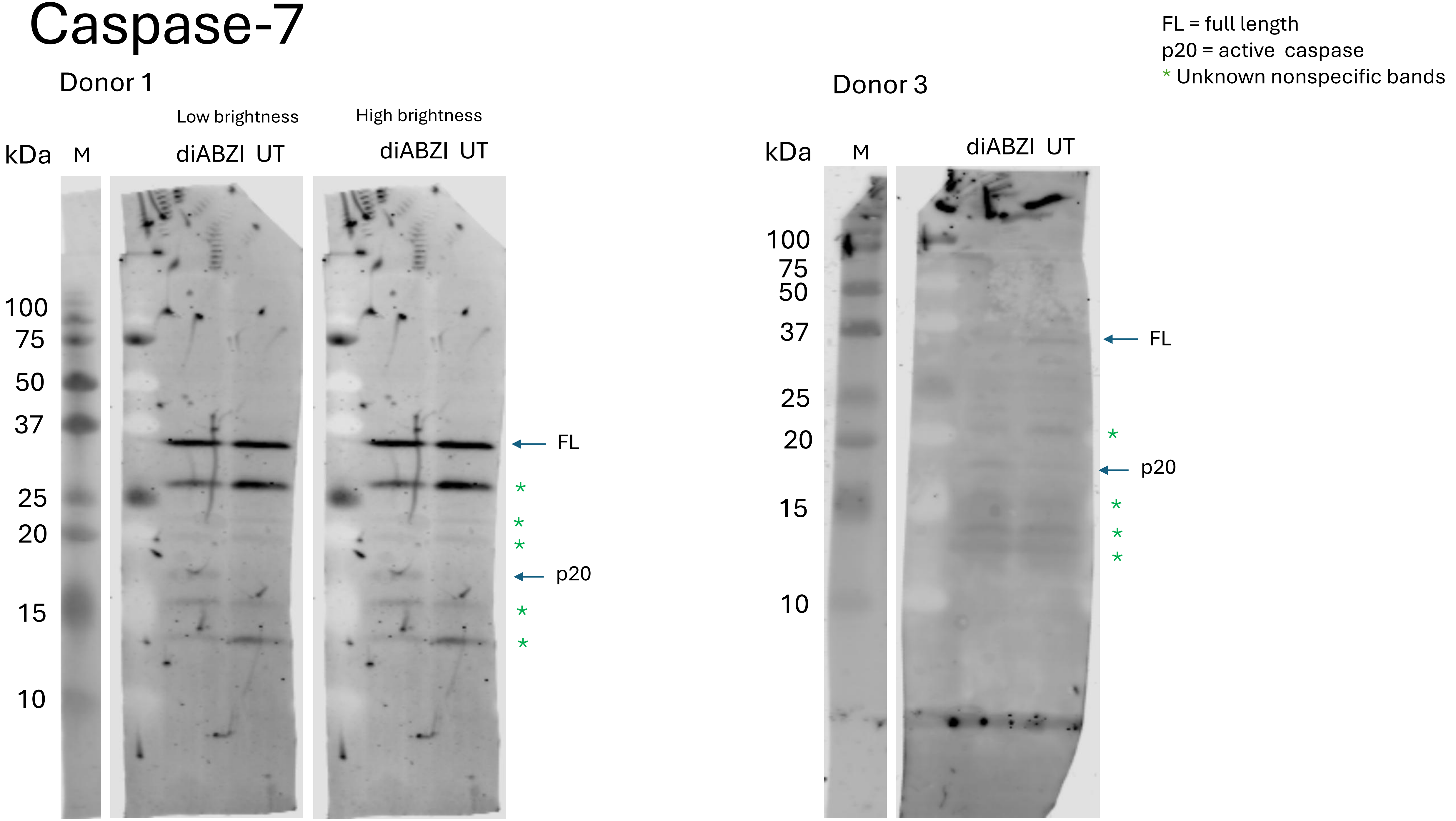
FL = full length
p20 = active caspase
* Unknown nonspecific bands
Donor 1
Low brightness
High brightness
kDa
M
diABZI UT
diABZI UT
100
75
50
37
25
20
15
10
FL
p20
Donor 3
kDa
M
diABZI UT
100
75
50
37
25
20
15
10
FL
p20

Caspase-8

FL = full length
p10 = active caspase
* Unknown nonspecific bands

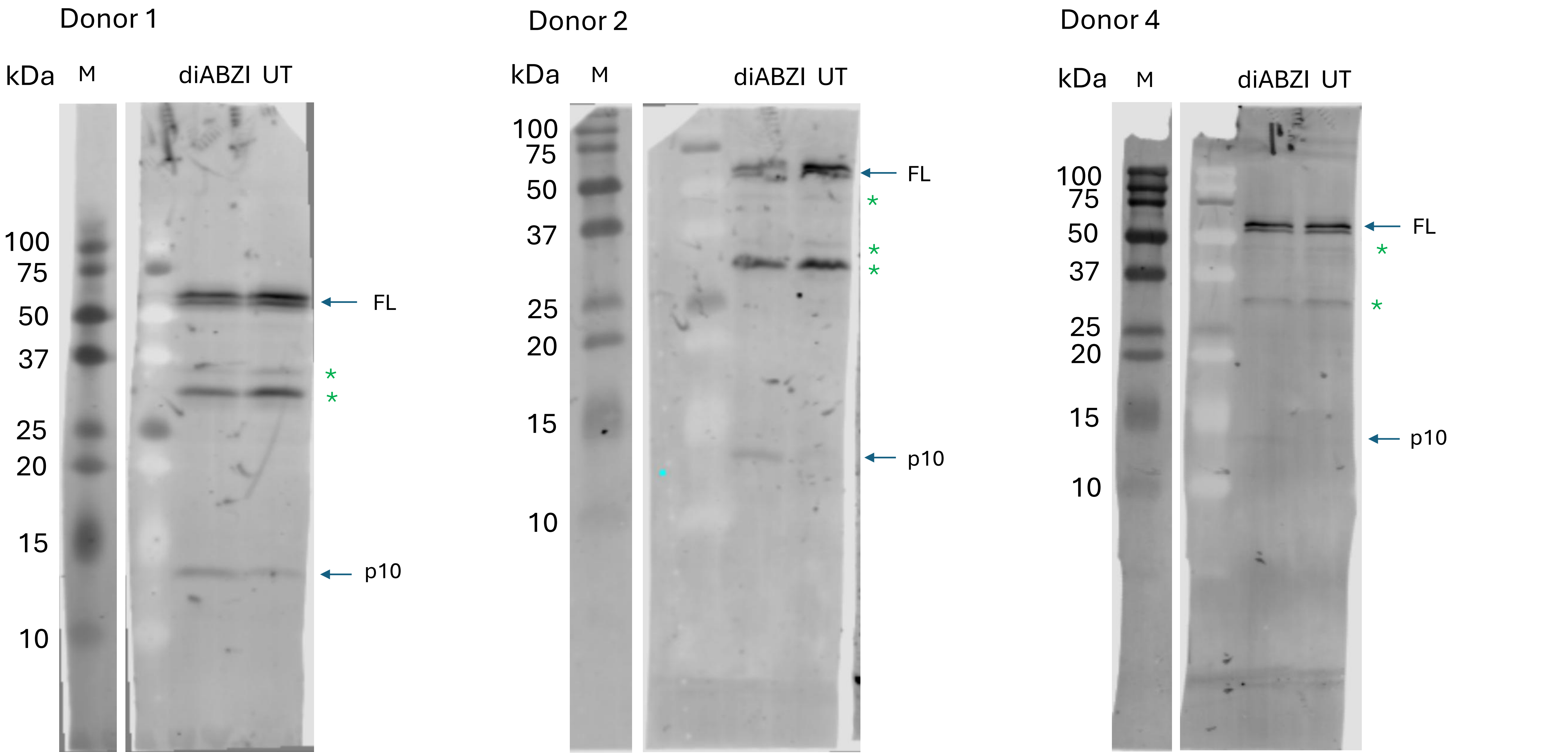

MLKL

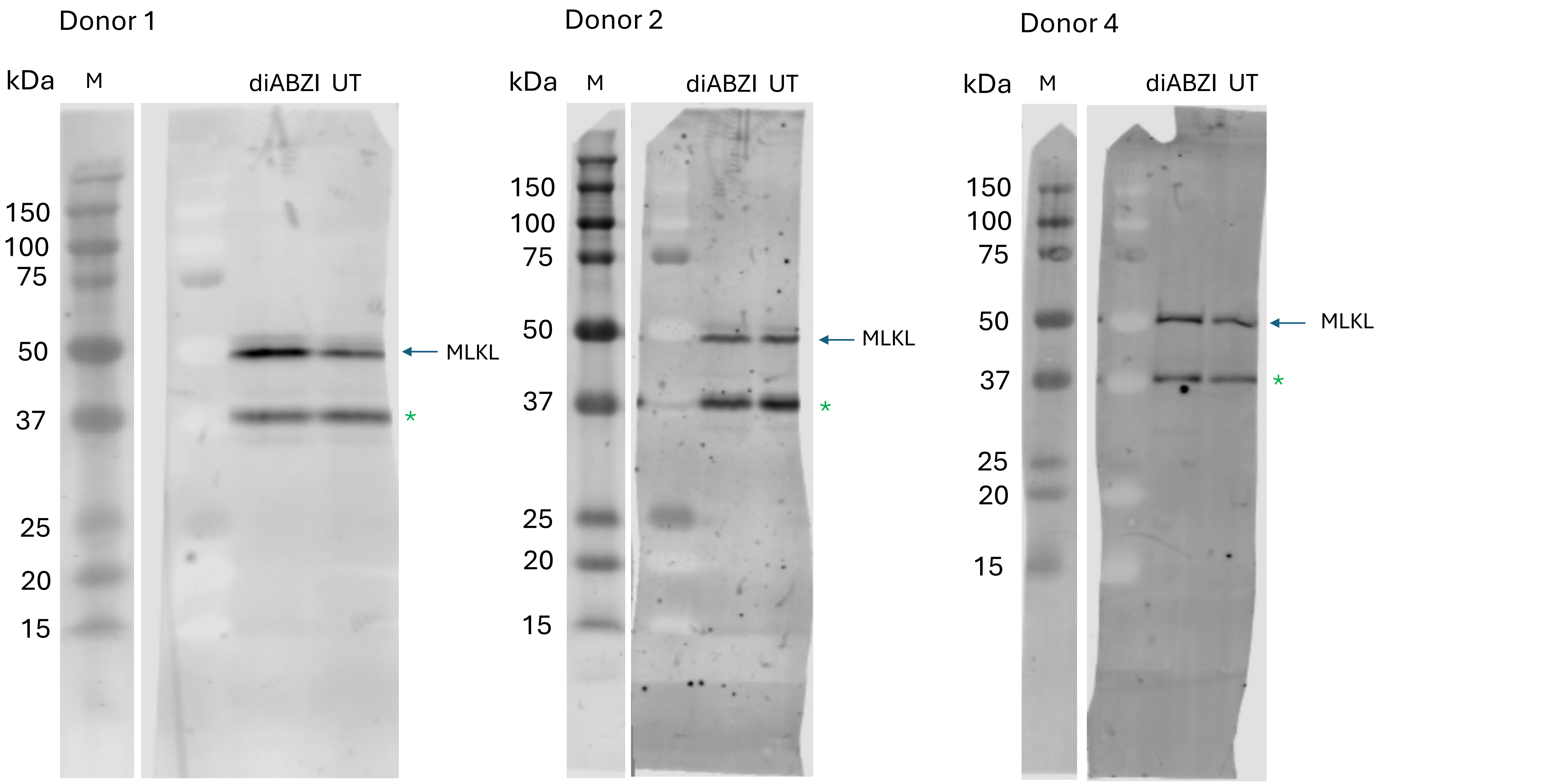
* Unknown nonspecific bands
Donor 1
kDa
M
diABZI UT
150
100
75
50
37
25
20
15
MLKL
*
Donor 2
kDa
M
diABZI UT
150
100
75
50
37
25
20
15
MLKL
*
Donor 4
kDa
M
diABZI UT
150
100
75
50
37
25
20
15
MLKL
*

# pMLKL

* Unknown nonspecific bands

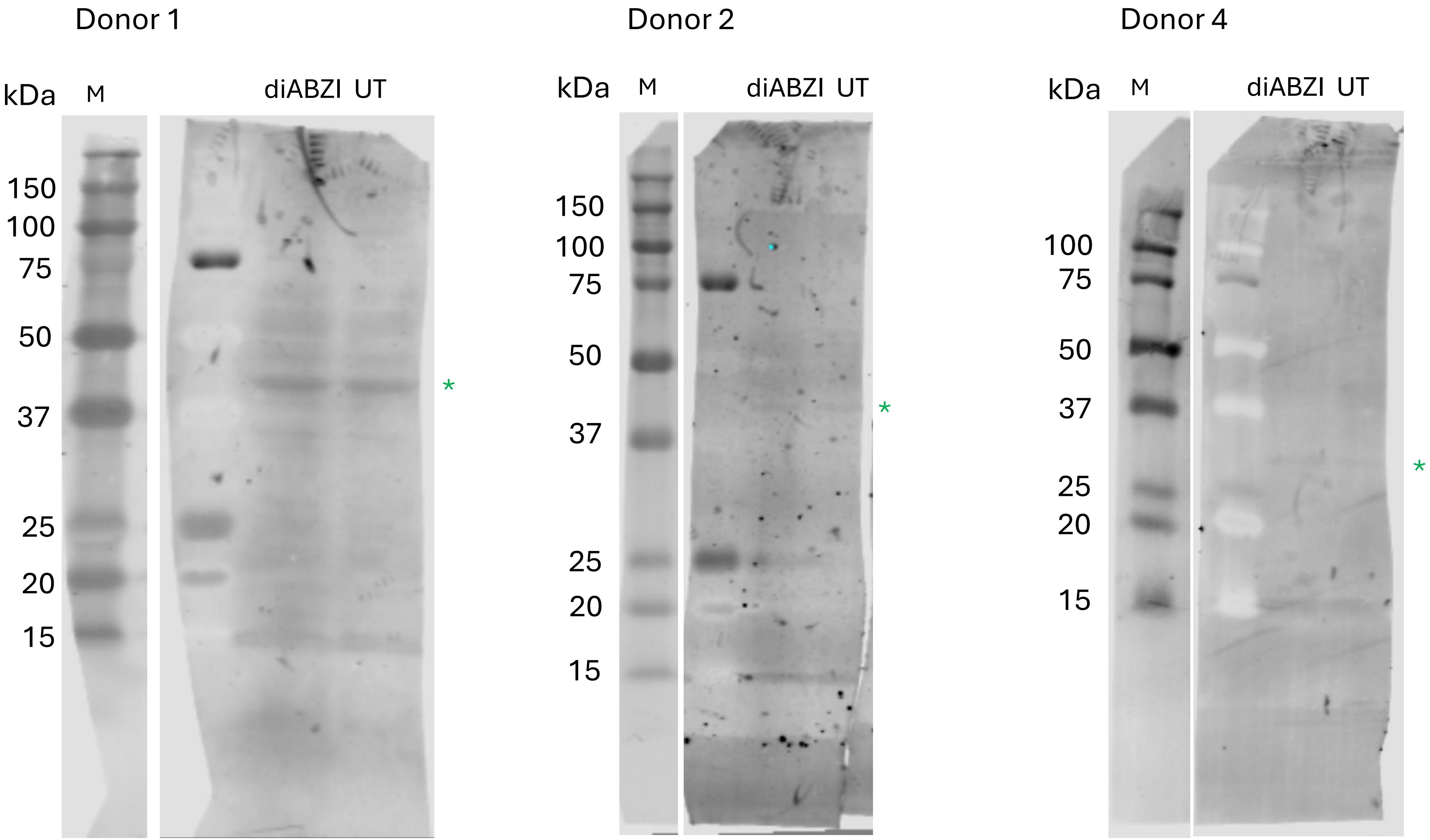

RIPK1

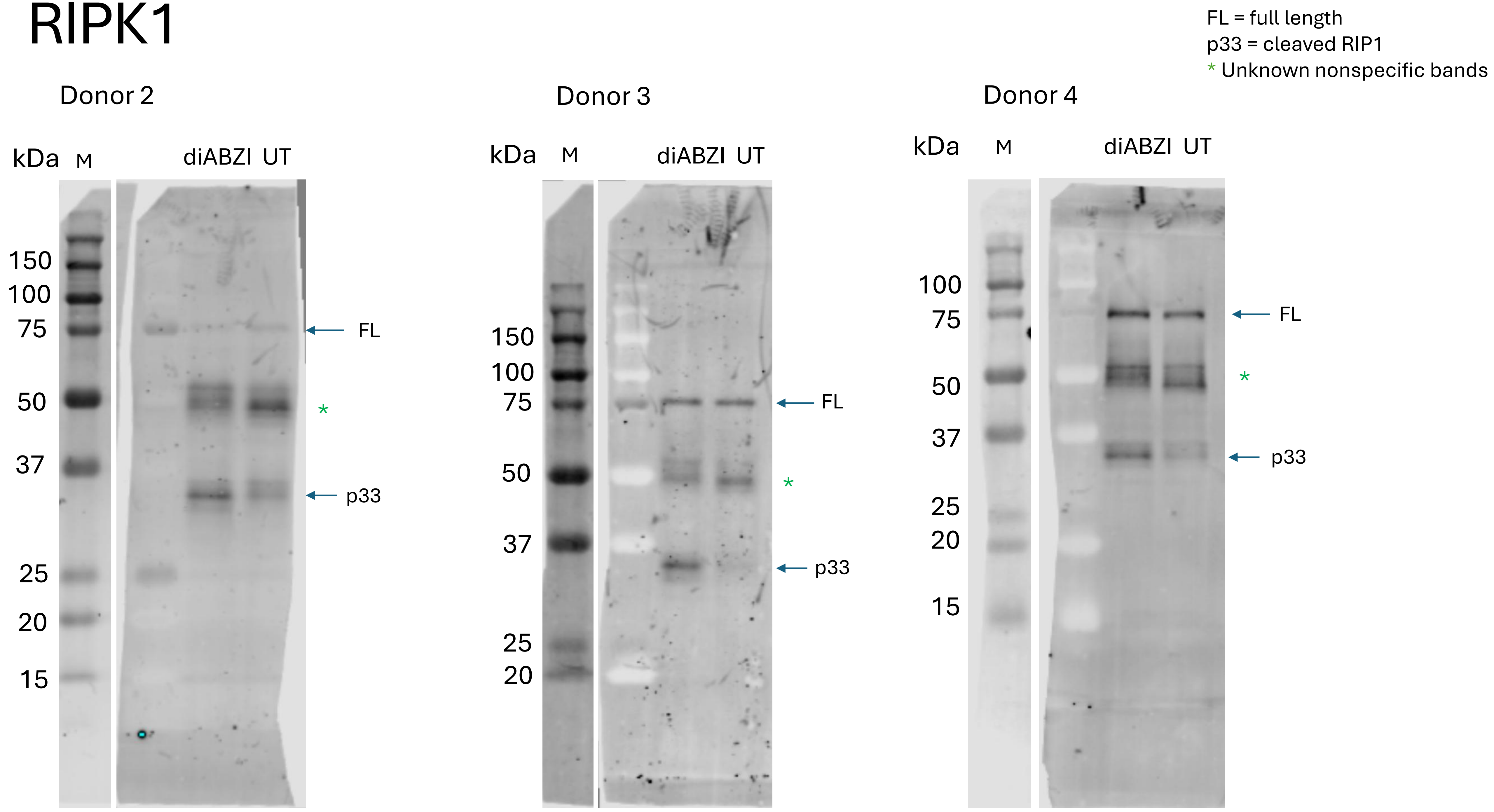

FL = full length
p33 = cleaved RIP1
* Unknown nonspecific bands
Donor 2
kDa
M
diABZI
UT
150
100
75
50
37
25
20
15
FL
*
p33
Donor 3
kDa
M
diABZI
UT
150
100
75
50
37
25
20
FL
*
p33
Donor 4
kDa
M
diABZI
UT
100
75
50
37
25
20
15
FL
*
p33

# pRIPK1

* Unknown nonspecific bands

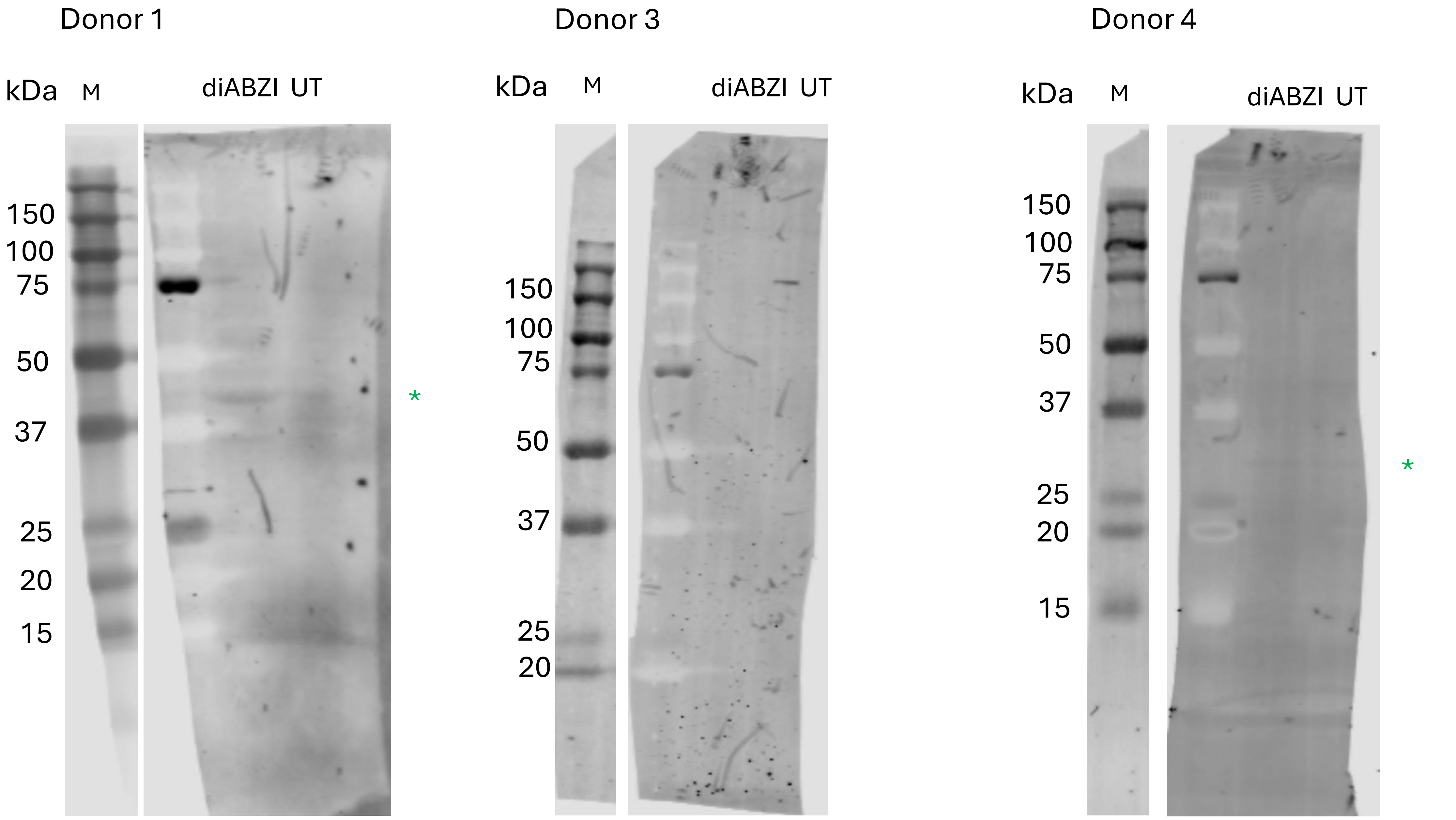
Donor 1
kDa
M
diABZI
UT
150
100
75
50
37
25
20
15
*
Donor 3
kDa
M
diABZI
UT
150
100
75
50
37
25
20
Donor 4
kDa
M
diABZI
UT
150
100
75
50
37
25
20
15
*

RIPK3

* Unknown nonspecific bands

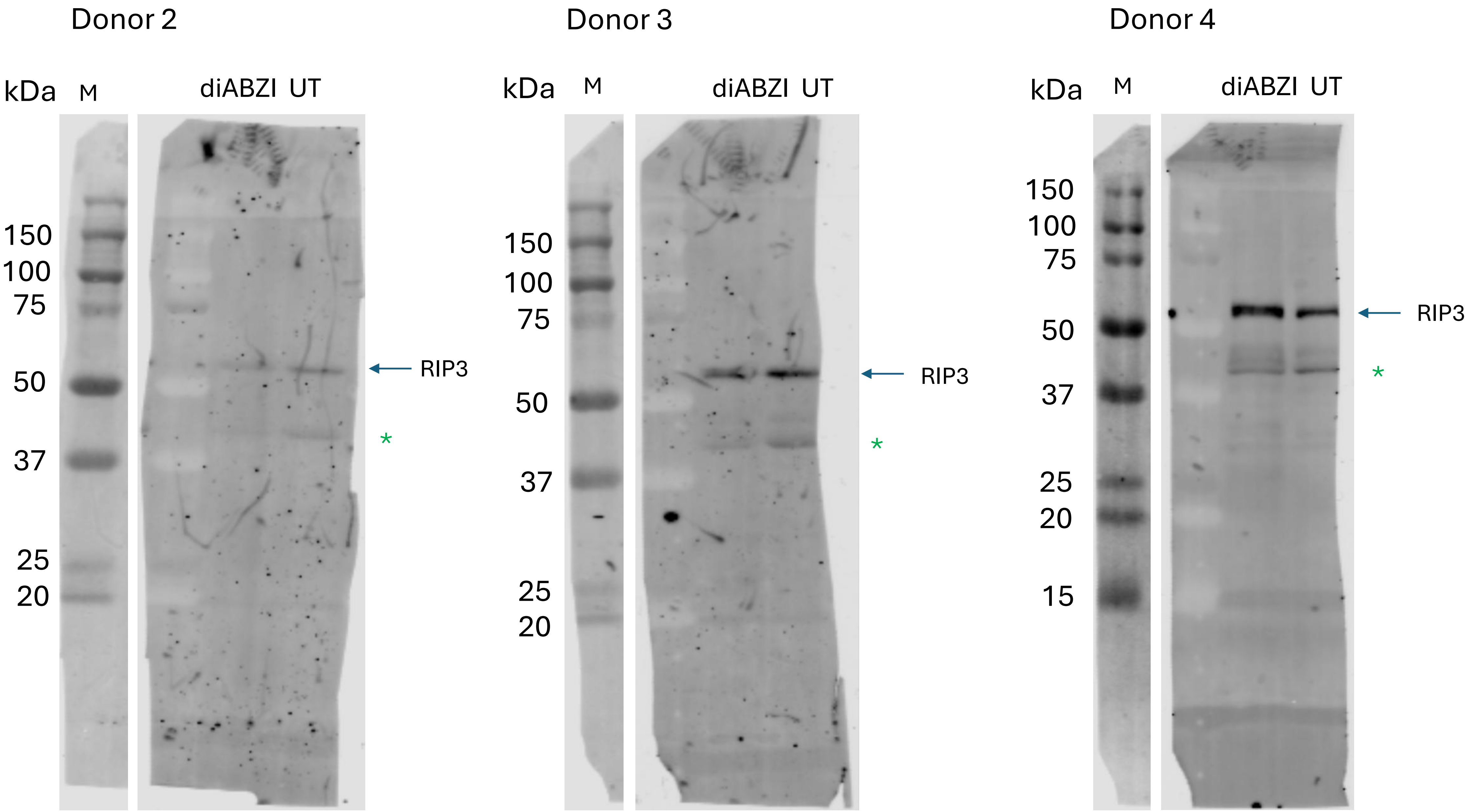

# pRIPK3

* Unknown nonspecific bands
* Unknown band present in treated samples, described by manufacturer: Abcam ab209384

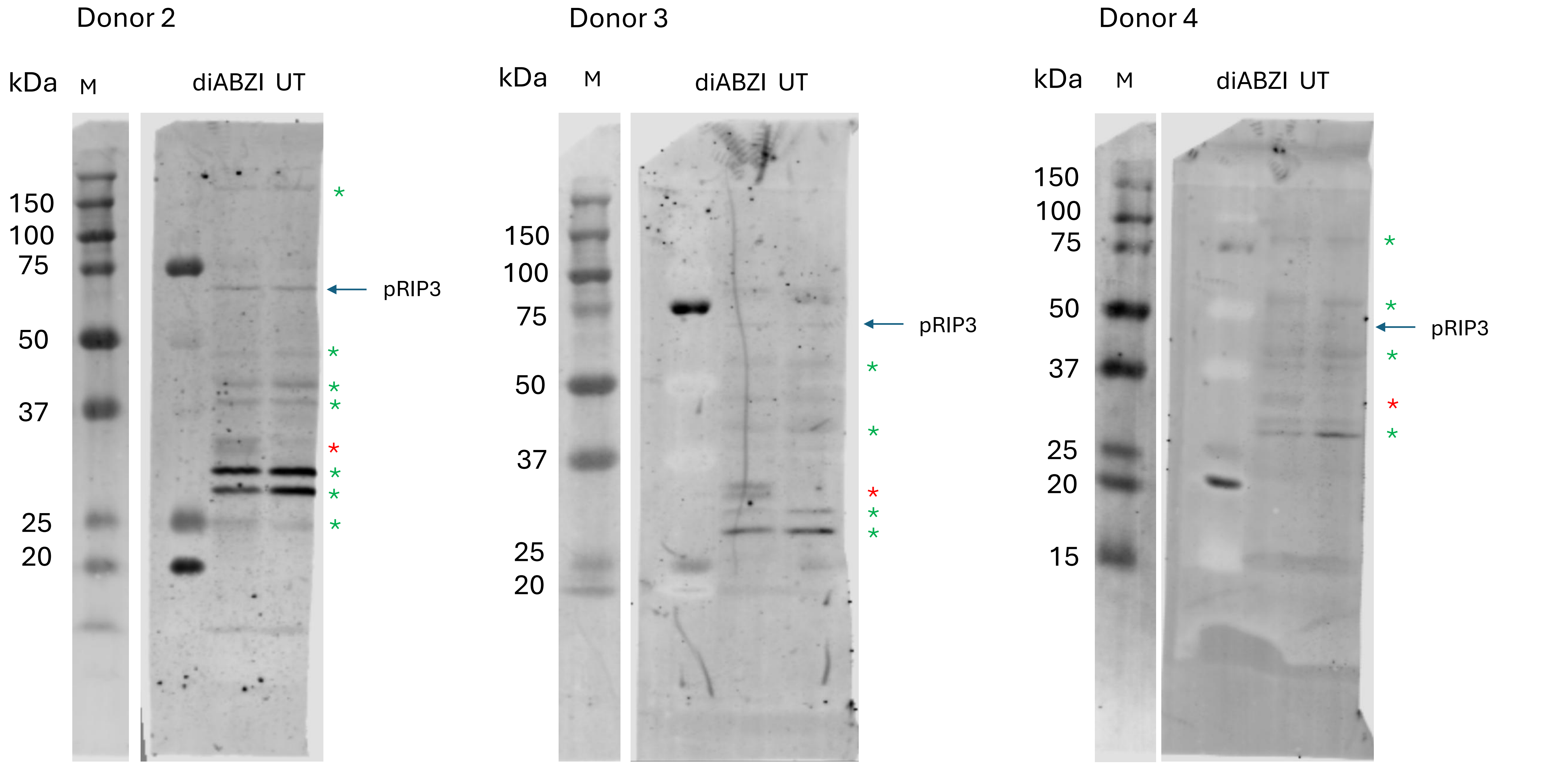

Supplement: Supplementary file 2 — WB-supplementary [file 41420_2025_2786_MOESM2_ESM.pdf]
